# Deep learning extracts MoA-specific signatures from high-throughput images of chemically and genetically perturbed *Corynebacteria*

**DOI:** 10.1126/sciadv.aeg6806

**Published:** 2026-09-25

**Authors:** Daniel Krentzel, Julienne Petit, Yves-Marie Boudehen, Nassim Mahtal, Elodie Sadowski, Agnès Zettor, Alexandra Aubry, Jeanne Chiaravalli, Nathalie Aulner, Stéphanie Petrella, Pedro M. Alzari, Christophe Zimmer, Anne Marie Wehenkel

**Affiliations:** ^1^ Institut Pasteur, Université Paris Cité, Imaging and Modeling Unit, F-75015 Paris, France.; ^2^ Institut Pasteur, Université Paris Cité, CNRS UMR 3528, Bacterial Cell Cycle Mechanisms Unit, F-75015 Paris, France.; ^3^ Institut Pasteur, Université Paris Cité, CNRS UMR 3528, Structural Microbiology Unit, F-75015 Paris, France.; ^4^ Institut Pasteur, Université Paris Cité, Photonic Bio-Imaging, Centre de Ressources et Recherches Technologiques (UTechS-PBI, C2RT), F-75015 Paris, France.; ^5^ AP-HP, Hôpital Pitié-Salpêtrière, Service de bactériologie, Centre National de Référence des mycobactéries et de la résistance des mycobactéries aux antituberculeux, F-75013 Paris, France.; ^6^ Institut Pasteur, Université Paris Cité, CNRS UMR 3523, Chemogenomic and Biological Screening Core Facility, C2RT, F-75015 Paris, France.; ^7^ Sorbonne Université, CNRS, Inserm, Centre d’Immunologie et des Maladies Infectieuses, CIMI, F-75013 Paris, France.; ^8^ Machine Biophotonics Lab, Rudolf Virchow Center for Integrative and Translational Bioimaging, University of Würzburg, Würzburg, Germany.; ^9^ Center for Artificial Intelligence and Data Science, University of Würzburg, Würzburg, Germany.

## Abstract

To address drug-resistant tuberculosis, the leading single-pathogen infectious killer, drugs with novel modes of action (MoAs) are urgently needed. Phenotypic screening of chemical libraries can identify antimicrobial compounds, but standard screens cannot reveal the MoA of hits, limiting targeted selection of compounds with novel MoAs. Here, we develop a deep learning (DL) model to screen drug-treated *Corynebacterium glutamicum* (*Cglu*), a surrogate for *Mycobacterium tuberculosis*. We train our DL model to distinguish between MoAs directly from high-throughput images. Our model robustly classifies MoAs of established antibiotics and recognizes the MoA of previously unseen antibiotics. Inhibitors with a previously unseen MoA cluster together and apart from reference drugs, enabling the detection of novel MoAs. Moreover, our model links images of chemical (drugs) and genetic (mutants) perturbations targeting similar pathways, supporting mutant-based target prediction of compounds with novel MoAs directly from images. Last, our DL model recovers known biological relationships from images alone using the *Cglu* cell cycle as a case study.

## INTRODUCTION

*Mycobacterium tuberculosis* (*Mtb*), a member of the suborder *Mycobacteriales* of *Actinobacteria*, is the causal agent of tuberculosis (TB). TB is among the top 10 causes of death worldwide and the leading cause of death in patients with HIV (*1*). In 2024, the World Health Organization revised its Bacterial Priority Pathogens List to add rifampicin-resistant *Mtb* (*2*). To face an increasing burden linked to antimicrobial resistance, there is an urgent need for novel treatments that overcome existing resistance mechanisms in *Mtb*. Now, TB is treated by administering a multidrug cocktail over various months with debilitating side effects. Carrying out this drug regimen is especially challenging in low-income countries and rural settings thereby hampering the ambitious goal to eradicate TB by 2050. Reaching this goal will require a deeper understanding of the complex *Mtb* biology to enable the identification and validation of new TB targets with the aim of discovering potent *Mtb* inhibitors preferably with novel modes of action (MoAs). This could not only enable the treatment of multi and extensively drug-resistant TB but also reduce the complexity and duration of current drug regimens.

Concerningly, the development of novel antibiotics has been in steady decline over the past three decades due to low success in validating drug candidates and limited commercial attractiveness despite considerable research and development costs (*3*, *4*). A key limitation of most drug discovery pipelines is the lack of efficiency and difficulty in reliably establishing the precise MoA of drug candidates (*4*–*6*). Phenotypic drug discovery (PDD) is an attractive avenue to discover compounds with novel MoAs (*7*). Its agnosticism to specific targets enables the discovery of previously unknown MoAs, and the use of live cells removes the need for optimizing chemical compounds to overcome cell permeability issues. The latter point is especially relevant for *Mycobacteriales*, since these bacteria have a unique and complex multilayered cell wall composed of three distinct macromolecules: peptidoglycan (PG), arabinogalactan (AG), and mycolic acids (*8*). These macromolecules form a formidable permeability barrier to most chemical compounds including potential antimicrobials (*9*).

In recent years, developments in image-based assays [e.g., Cell Painting (*10*)] and image analysis methods [e.g., CellProfiler (*11*)] that quantitatively assess cellular phenotypic changes induced by molecules have led to an explosion in image-based PDD screens (*12*). Instead of relying on mono-parametric readouts like optical density (OD), these methods enable a holistic assessment of both cellular and subcellular changes by extracting hand-crafted features from segmented cells. This allows the identification of compounds with subtle effects which can then be further optimized by medicinal chemists. These image-based screens address a key limitation of growth inhibition assays, namely, MoA recognition. Successful MoA recognition of molecules from images alone has been shown in various eukaryotic screens and pioneering work by Nonejuie *et al.* (*13*) and Zoffmann *et al.* (*14*) has demonstrated that such methods can also be applied to bacterial screens. For instance, in a recent image-based phenotypic screen of *Acinetobacter baumannii*, such an approach was crucial to identify a novel class of antibiotics targeting the lipopolysaccharide transport machinery (*15*).

Directly applying these approaches to screen for TB drugs is, however, challenging, since *Mtb* exhibits a high degree of cell-to-cell variability even in the absence of drug exposure. Prior work has thus sought to account for the heterogeneous response of individual *Mtb* cells either by specifically defining hand-crafted features that account for this cell-to-cell variability (*16*) or by training a type of deep learning (DL) model called a convolutional neural network (CNN) to predict drug MoAs from images of drug-treated *Mtb*, thereby circumventing the need for cell segmentation and subsequent hand-crafted feature extraction (*17*). More recently, integration of multimodal data using autoencoders has also been used to dissect mechanisms of action of TB drugs (*18*). While these methods enable MoA recognition of hit compounds with respect to known compounds, they are unable to identify drug targets of active compounds with novel MoAs. A promising avenue to overcome this limitation is to assume that drug treatments and genetic mutations result in similar phenotypes if they affect the same proteins or pathways. Along these lines, prior work has shown that hand-crafted features obtained from images of CRISPRi *Mycobacterium smegmatis* (a nonpathogenic surrogate model of *Mtb*) mutants and drug-treated bacteria tend to cluster according to the targeted pathways (*19*).

Using nonpathogenic surrogate models of *Mtb*, like *M. smegmatis*, to study the effect of TB drugs can markedly speed up and simplify experimental protocols, since *Mtb* is highly pathogenic, which requires experiments to be carried out within high biosafety level (BSL-3) laboratories, and slow growing (24-hour doubling time) thus limiting throughput. However, the genome of *M. smegmatis* is almost two times larger than that of *Mtb* (7 and 4.4 Mbp, respectively) with larger functional redundancy, making the study of specific cellular pathways more challenging. We therefore use another nonpathogenic surrogate model for *Mtb* called *Corynebacterium glutamicum* (*Cglu*), a member of the *Mycobacteriales*, with a short doubling time of about 1 hour and a genome size comparable to that of *Mtb* (3 Mbp). Moreover, *Cglu* and *Mtb* share common core mechanisms related to cell division and cell wall synthesis, as well as a complex multilayered cell wall (*20*, *21*). A key advantage of *Cglu* is its ability to withstand the loss of its outer cell envelope layers, thereby enabling the study of its biosynthetic proteins (*22*) which are potential drug targets. For the context of a TB drug screen, *Cglu* has been shown to respond to antibiotics and particularly to TB drugs that target core conserved mechanisms (*23*, *24*). However, this model will only be valid on conserved core mechanisms and not on *Mtb* specific mechanisms.

Here, we present an image-based high-throughput TB drug discovery pipeline that links drug-treated and mutated *Cglu* strains where the same or a similar pathway has been disrupted. To that end, we acquire high-throughput images of *Cglu* exposed to several known TB drugs and use these images to train a custom CNN for the task of predicting antibiotic MoAs from images alone. We show that our model successfully predicts targets of noncanonical TB drugs and distinguishes compounds with previously unseen MoAs. We furthermore demonstrate that our DL model can extract meaningful phenotypic signatures from images of drug-treated bacteria and mutants in which the same pathway has been disrupted. This allows for the association of genetically and chemically induced phenotypic changes directly from images paving the way toward mutant-based target deconvolution. Last, we show that our DL model provides insights into the function of genes affecting the cell cycle by clustering images of mutants belonging to similar pathways. Together, our approach presents a promising avenue for image-based phenotypic screening in bacteria, mutant-based target deconvolution of novel compounds and investigating the role of bacterial genes directly from images.

## RESULTS

### Bacterial high-throughput image acquisition

To obtain a dataset for training and testing our DL model, we acquired high-throughput images of a *Cglu* strain (ATCC13032) grown in controlled medium and exposed bacteria to 18 established drugs with five different MoAs associated with seven different biological pathways (Table 1). Drugs were grouped according to modes rather than mechanisms of action, since not all drugs have a clearly identified and/or unique protein target. To train our DL model, we selected a subset of these antibiotics belonging to five different MoAs: (i) ethambutol inhibits the arabinosyltransferases involved in the synthesis of the AG layer, a critical component of the mycobacterial cell envelope (*25*, *26*); (ii) amoxicillin, carbenicillin and cefotaxime are beta-lactam compounds that target penicillin-binding proteins (PBPs) involved in PG synthesis (*27*); (iii) the two fluoroquinolones (FQs) ciprofloxacin and moxifloxacin and the aminocoumarin novobiocin inhibit DNA gyrase—the sole topoisomerase in *Cglu* responsible for regulating DNA supercoiling and decatenating daughter chromosomes (*28*); (iv) clarithromycin, doxycycline and linezolid target the ribosome (*29*); and (v) clofazimine which is thought to target the respiratory chain and has also been shown to affect membrane homeostasis in *Mtb* (*30*) and *Staphylococcus aureus* (*31*).

**Table 1.** Modes of action of antibiotics from training and testing dataset.

Mode of action	Antibiotic	Training data	Time and concentration experiment	MIC (μM)	References
Cell wall (PBP)	Amoxicillin	✓	✓	0.34	(*55*)
	Carbenicillin	✓	✓	0.66	This work
	Cefotaxime	✓	✓	0.26	(*55*)
	Ampicillin			0.36	This work
Cell wall (AG)	Ethambutol	✓		4.90	This work
DNA gyrase	Ciprofloxacin	✓	✓	0.76	(*23*)
	Moxifloxacin	✓	✓	0.30	(*35*)
	Novobiocin	✓		26	(*35*)
	Gepotidacin			0.45	(*23*)
	BDM71403			0.27	(*23*)
Ribosome	Clarithromycin	✓		1.34	This work
	Doxycycline	✓	✓	0.27	This work
	Linezolid	✓		2.96	This work
Cell membrane	Clofazimine	✓		2.11	This work
RNA polymerase	Rifampicin		✓	0.15	This work
	Rifabutin		✓	0.30	This work
Folate synthesis	Trimethoprim			>64	This work
	Sulfamethoxazole			>64	This work

**Fig. 1.**
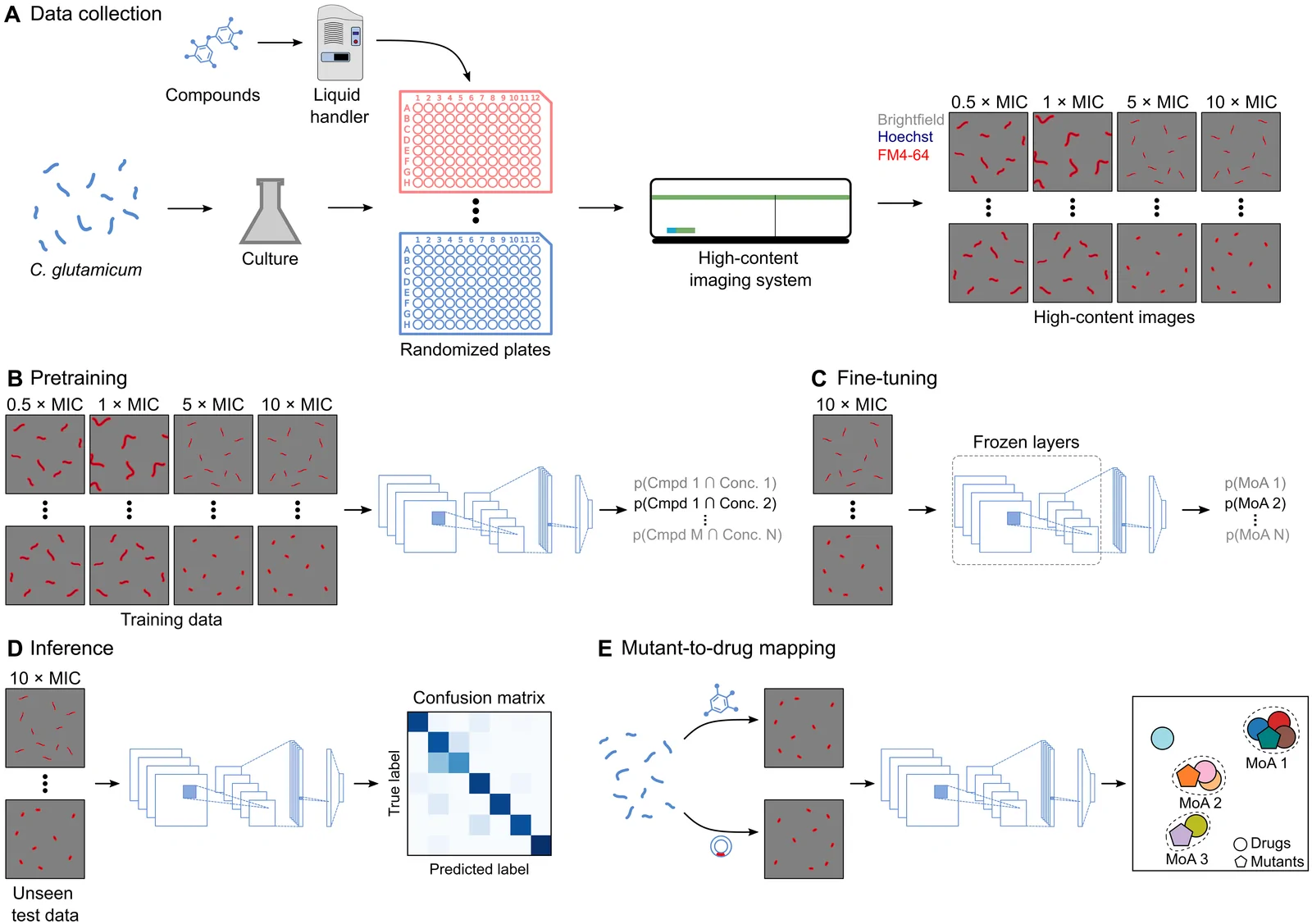
Dataset acquisition and model training. (**A**) Antibiotic compounds were distributed with randomized plate layouts at four different concentrations (0.5× MIC, 1× MIC, 5× MIC, and 10× MIC) onto six 96-well plates with an acoustic liquid handler. *Cglu* cultures were grown for 16 hours in six biological replicates and incubated with antibiotics on the previously prepared 96-well plates. Bacteria were then fixed with ethanol, stained with FM4-64 (cell membrane) and Hoechst 33342 (DNA), and imaged on a high-content microscope with a 63× water-immersion objective in three channels (brightfield, FM4-64, and Hoechst 33342). (**B**) High-throughput images across all concentrations from five of the six plates were used to pretrain a custom DL model to predict the combination of antibiotic and concentration from images alone. (**C**) After pretraining, convolutional layers were frozen, and images of bacteria exposed to antibiotics at 10× MIC were used to fine-tune the fully connected layers. During fine-tuning, we used a triplet loss to explicitly promote the formation of clusters by MoA in latent space, a center loss to increase tightness of clusters, and a weighted cross-entropy loss for prediction. (**D**) Performance quantification and further analysis were conducted on unseen images from a hold-out test plate to ensure robustness to biological and technical variation. (**E**) Images of mutants and drugs were then jointly embedded using the previously trained DL model to link chemical and genetic perturbations by pathway.

For each of the above-mentioned drugs, we determined or used published minimum inhibitory concentrations (MICs) (Table 1 and Materials and Methods) and exposed bacteria to four different concentrations (0.5, 1, 5, and 10× MIC). Drugs were distributed in a random layout onto six 96-well plates with an acoustic liquid handler (Beckman Coulter Echo 550) to decorrelate conditions from well-plate position (fig. S1). We exposed bacteria to drugs for 16 hours starting in early exponential phase. Bacteria were stained for membrane (FM4-64) and nucleoid (Hoechst 33342) and fixed with ethanol. To ensure consistent densities between treatment conditions, OD measurements were used to normalize the number of bacteria in each well before transferring them to imaging plates. Then, 50 three-channel (brightfield, FM4-64, and Hoechst 33342) images per condition (i.e., combination of concentration and antibiotic) were acquired on a high-content screening system (Revvity Opera Phenix Plus) in widefield mode with a 63× water-immersion objective. In total, six biological replicates were acquired on six separate plates (Fig. 1A).

### Model architecture and training strategy

To obtain high-dimensional feature vectors from bacterial high-throughput images in an end-to-end fashion, we modified a DL model that we previously developed to recognize the MoA of antibiotics from images of *Escherichia coli* (*32*)*.* First, we pretrained our model using five of the six available plates (biological replicates) to predict combinations of drugs and concentrations directly from images as described in our previous work (Fig. 1B and Materials and Methods) (*32*). Then, we added a fine-tuning step where the convolutional layers were frozen and only parameters in the fully connected layers of our model were updated (Fig. 1C and Materials and Methods). For fine-tuning, we directly trained our model to predict drug MoAs using images of bacteria exposed to antibiotics for 16 hours at the highest concentration (10× MIC) to increase the likelihood of bacteria showing discriminative phenotypes. We found that the addition of this fine-tuning step during training improved clustering by MoA (fig. S2). We then tested the performance of our DL model on a sixth hold-out test plate (biological replicate) with a different randomized plate layout (fig. S1) to ensure robustness to positional effects and biological variation. To compare phenotypes between different perturbations, we obtained high-dimensional feature vectors from the penultimate layer of our model (Fig. 1D).

To verify that our choice of incubation time (16 hours) and concentration (10× MIC) did not adversely affect MoA classification performance, we acquired a subset of eight antibiotics (Table 1) belonging to four distinct MoAs (PBP, DNA gyrase, ribosome, and RNA polymerase) at both higher (50 and 100× MIC) and lower concentrations (0.5, 1, and 5× MIC) using two time points (6 and 16 hours) in triplicates (Materials and Methods). We normalized cell densities before imaging to ensure that bacteria were present at equivalent concentrations (fig. S3, A and B) and then fine-tuned our model on each of these combinations whereafter we computed MoA classification accuracies on three rotations of training and test replicates (fig. S3C). We found that mean MoA classification accuracies were consistently high (between 97.2 and 99.8%) as the concentration reached 5× MIC regardless of incubation time. We observed that MoA classification performance improved with longer incubation time at the two lowest concentrations, indicating that bacterial phenotypes become more specific for a given MoA as incubation time and concentration increase.

To explore whether we could directly link chemical to genetic perturbations from images, we prepared a third dataset based on biological triplicates of plates containing seven mutant strains and *Cglu* bacteria exposed to seven different antibiotics (Table 2 and Materials and Methods). After acquiring high-throughput images as described above, we used the DL model, which we previously trained to predict the MoA of antibiotics outlined in Table 1, to obtain feature vectors for all conditions which we then used to directly link drug perturbations to bacterial mutant strains (Fig. 1E).

**Table 2.** Description of dataset of disrupted pathways of mutants and drugs. Description of dataset shown in Fig. 4 of disrupted pathways of mutants and drugs. Full mutant description can be found in table S1.

Mode of action	Mutant name	Drugs
DNA segregation	*gyrA*	BDM71403
		Ciprofloxacin
	*gyrB*	Gepotidacin
		Moxifloxacin
Cell wall/division defect	*sepF*	Ampicillin
	Δ*glp*	
	Δ*glpR*	Carbenicillin
	Δ*steAB*	
Cell wall/elongation defect	*wag31*	Ethambutol

### DL identifies the MoA of previously seen drugs with high accuracy directly from images

**Fig. 2.**
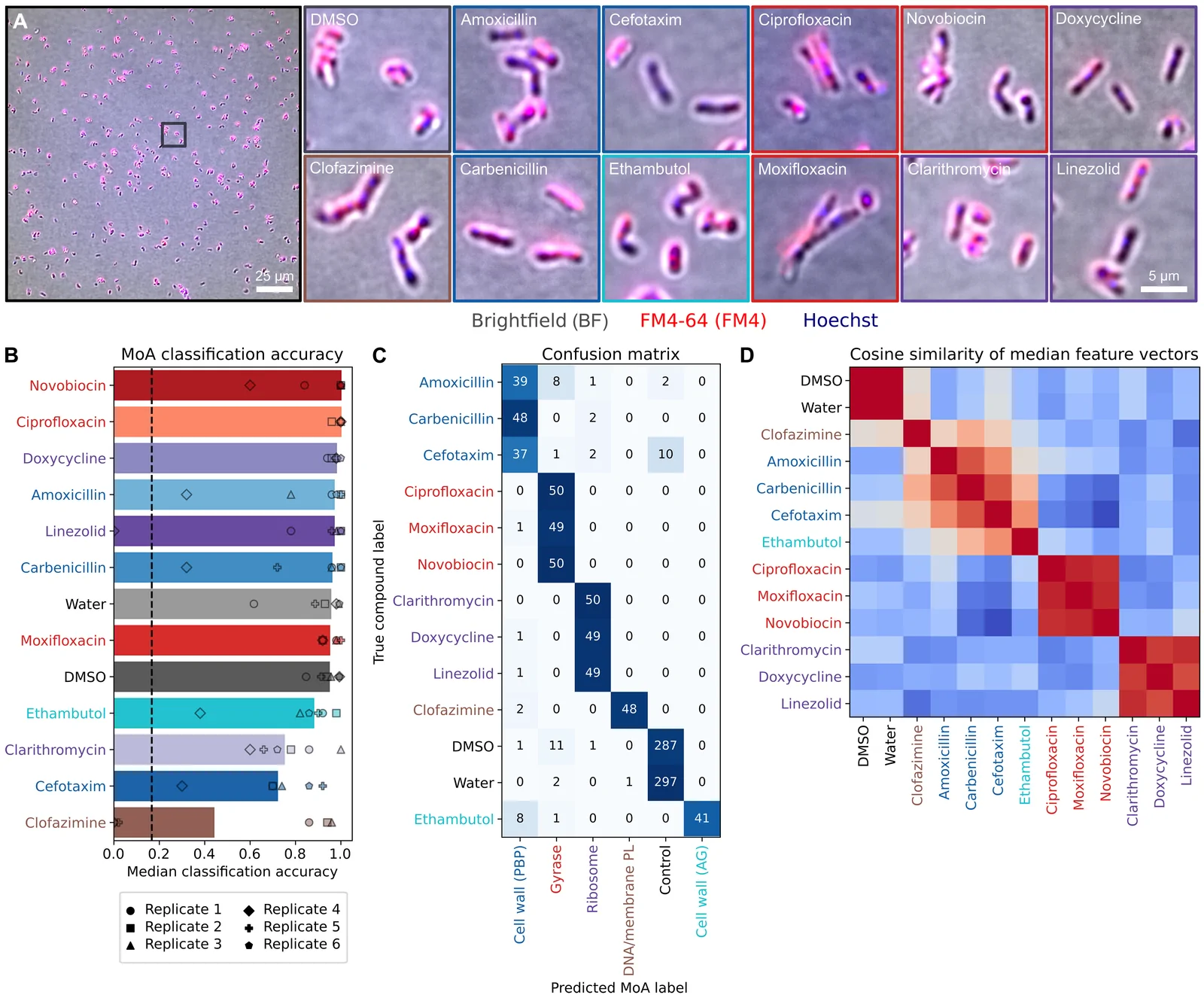
DL recognizes MoAs of antibiotics from images alone. (**A**) Representative example images of *Cglu* bacteria exposed to antibiotics at 10× MIC and acquired on a high-content microscope with a 63× water-immersion objective are shown with scale bars. Brightfield is shown in grayscale, cell membrane (FM4-64) in red, and DNA (Hoechst 33342) in blue. (**B**) Median MoA classification accuracies shown by compound and hold-out test replicate (biological and technical replicates). (**C**) Confusion matrix obtained from a DL model trained on three-channel images (brightfield, FM4-64, and Hoechst 33342) of bacteria exposed to antibiotics at 10× MIC and tested on unseen images from a hold-out test plate. (**D**) Cosine similarity computed on median feature vectors obtained from images of drug-treated bacteria shows high similarity (in red) of antibiotics by MoA.

We first validated that our DL model could distinguish the MoA of established drugs. To that end, we used high-throughput images of *Cglu* bacteria exposed to 11 antibiotics, most of which are used in clinics to treat TB (Table 1). We used the DL model to predict the MoA from images of bacteria exposed to antibiotics at 10× MIC (Fig. 2A) on six rotations of the training and testing plates on the subset of the 11 antibiotics that were included in the training set. Our model achieved a per–field of view (FOV) classification accuracy of 87.25 ± 7.27% and a well-level MoA classification accuracy of 92.75 ± 8.93% cross-validated across the six hold-out test replicates (Fig. 2, B and C). Despite these high average classification accuracies, we observed a clear split in prediction accuracy for clofazimine. On three of the six plates (replicates 1, 2, and 3), clofazimine was correctly classified as inhibiting the respiratory chain/membrane homeostasis, whereas on the three remaining plates (replicates 4, 5, and 6), most images were classified as controls. Upon visual inspection of the images (fig. S4), we found that cell morphologies were consistently elongated on the first three plates. Conversely, cell morphologies were wild type (WT)–like on the last three plates consistent with these images being classified as controls by our DL model (fig. S4, E to G). We speculate that the lack of phenotypes could either be due to compound instability (plates 4 to 6 were acquired multiple days after the initial plate) or due to insufficient compound transfer during nanoliter-scale dispensing.

Next, we compared our DL approach to segmentation-based profiling by first extracting 528 hand-crafted features from the images with a commercial software [Revvity, Signals Image Artist (SImA)] and then training a random forest classifier to predict the MoA on six rotations of training and test plates (Materials and Methods and fig. S5A). We found that the random forest classifier achieved a significantly lower (*P* = 0.031, Wilcoxon signed-rank test) well-level MoA classification accuracy of 72.46 ± 8.57% compared to our DL model (92.75 ± 8.93%) on the 11 previously seen antibiotics.

To verify that the DL model learned to extract more similar feature vectors for drugs belonging to the same MoA, we computed a cosine similarity matrix using the median feature vectors obtained for each drug treatment (Fig. 2D). As expected, we found that compounds with similar MoAs exhibited high cosine similarity. Despite training the DL model to consider clofazimine and ethambutol as distinct classes, median feature vectors for both conditions exhibited similarity to drugs targeting PBPs. Cells treated with clofazimine exhibit an elongated cell length similar to beta-lactam inhibition (fig. S6). This indicates that our DL model learned to pick up common phenotypic features related to global cell envelope perturbations while also encoding discriminative features associated with individual MoAs, namely, disruption of PG, AG, and membrane homeostasis.

Last, we investigated the robustness of the DL model to changes in concentration using images of bacteria exposed to three concentrations below 10× MIC (0.5, 1, and 5× MIC). At the lowest concentration (0.5× MIC), 10 of the 14 drugs with a previously seen MoA were classified as control (fig. S7A). The remaining drugs—three PBP inhibitors (carbenicillin, cefotaxime, and ampicillin) and one DNA gyrase inhibitor (novobiocin)—were correctly classified regardless of concentration, suggesting that they induce strong MoA-specific phenotypic changes even at low concentrations. At a concentration of 5× MIC, 13 of the 14 drugs with a previously seen MoA were correctly classified, suggesting that drug phenotypes stabilized. The exception was clofazimine which was classified as a PBP inhibitor at 5× MIC (in line with the high cosine similarity observed between clofazimine and PBP inhibitors at higher concentrations in Fig. 2D). We also observed that compounds belonging to the same MoA followed similar dose-dependent trajectories in feature space (fig. S7, B and C).

Together, these results show that our DL model can robustly classify drugs by their MoA from images alone and recognizes similarities between drugs targeting the same or related pathways. Moreover, MoA predictions above 5× MIC are robust, which indicates that accurate MoA determination of putative hit compounds could be feasible at a single concentration directly from high-throughput images.

### DL identifies MoAs of previously unseen drugs and detects novel MoAs

Having verified the ability of our DL model to correctly recognize the MoA of drugs seen during training, we wanted to check its performance on unseen candidate drugs using the previously seen antibiotics as reference compounds (Table 1). We picked candidate drugs from three different categories: (i) compounds which exhibit no detectable activity; (ii) active compounds where the MoA, but not the drug, was present in the training data; and (iii) active compounds with a previously unseen MoA. For the first category, we selected trimethoprim and sulfamethoxazole—two folic acid synthesis inhibitors that have bacteriostatic effects and are typically administered in combination (*33*, *34*) and are inactive in *Cglu* at the concentrations that we tested (fig. S8). For the second category, we used three compounds that are relevant for *Mycobacteriales* species but not typically used to treat TB infections in clinics: two novel bacterial topoisomerase inhibitors (NBTIs) targeting the DNA gyrase [gepotidacin and BDM71403 (*35*)] and one drug targeting PBPs (ampicillin). NBTIs differ from FQs in their mechanism of action through a distinct topoisomerases binding mode: While two FQ molecules intercalate into the cleaved DNA and interact with the GyrB and GyrA subunits to block the DNA religation step, a single NBTI molecule intercalates into the single-stranded cleaved DNA and binds only to GyrA, trapping the gyrase in a DNA-bound conformation (*28*). For the third category, we chose rifampicin and rifabutin which target the RNA polymerase.

**Fig. 3.**
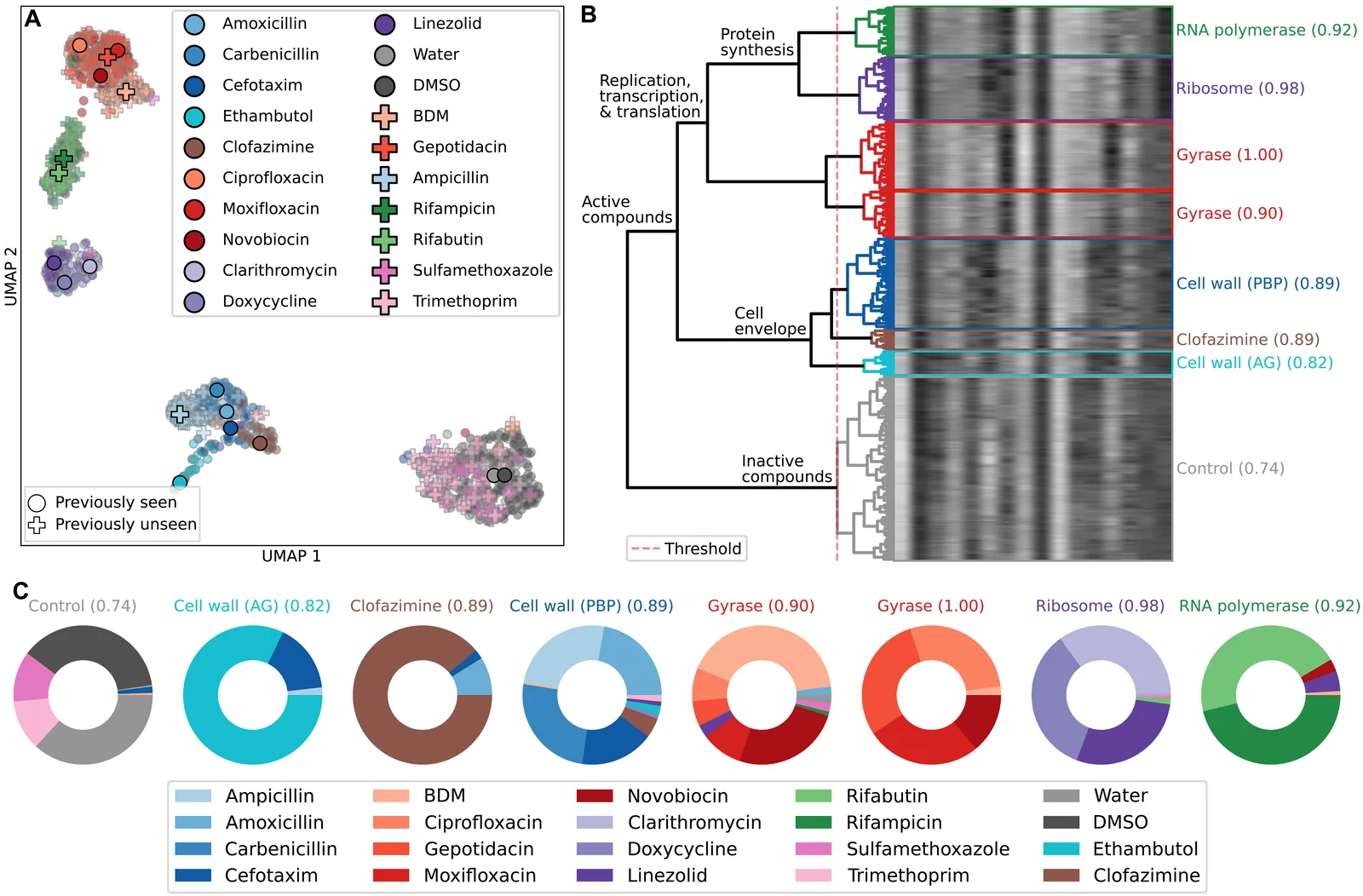
DL correctly identifies drug targets of previously unseen compounds. (**A**) UMAP of feature vectors with previously seen drugs shown as dots and previously unseen drugs with crosses. Large dots/crosses correspond to median feature vectors. Four of the previously unseen drugs belong to two previously unseen MoAs inhibiting folic acid synthesis and RNA polymerase. Sulfamethoxazole and trimethoprim (folic acid synthesis) were included as negative controls, since these drugs need to be administered in combination for effects to be observed. (**B**) Hierarchical clustering on feature vectors with the threshold set such that negative controls (water and DMSO) cluster. Clusters are annotated by the most common MoA with the proportion of drugs belonging to a given MoA shown in brackets. (**C**) Pie charts showing the composition of each cluster by drug with colors corresponding to the drug identity.

First, we used our DL model to obtain feature vectors of these previously unseen candidate drugs at 10× MIC and jointly visualized them on a UMAP plot with feature vectors of reference drugs that were previously seen during training (Fig. 3A). We found that candidate drugs with a previously seen MoA clustered tightly with reference drugs belonging to the same MoA. For instance, gepotidacin and BDM71403 correctly clustered with DNA gyrase inhibitors, while ampicillin clustered with the other beta-lactams. The two inactive drugs trimethoprim and sulfamethoxazole colocalized with negative controls, while rifampicin and rifabutin—the two drugs with a previously unseen MoA—formed a separate cluster.

To further quantify these observations, we performed hierarchical clustering (Materials and Methods) on feature vectors (Fig. 3B). The negative control feature vectors [water and dimethyl sulfoxide (DMSO)] were used to define the clustering threshold. We observed two major clusters consisting of inactive and active compounds. The latter was in turn composed of two groups, one encompassing drugs disrupting replication, transcription, and translation, and the other including drugs targeting the cell envelope, in agreement with the UMAP representation shown in Fig. 3A. The replication, transcription, and translation cluster was further split into compounds targeting protein synthesis and DNA gyrase. Within the protein synthesis cluster, the two RNA polymerase inhibitors formed a tight and clearly separated cluster (Fig. 3C) consistent with our observations from the UMAP visualization in Fig. 3A. This indicates that the alterations in bacterial phenotypes resulting from drug exposure mirror functional similarities in the pathways they affect.

Then, we used our DL model to obtain MoA predictions from candidate drugs (fig. S9A). Images of candidate drugs where the MoA was included in the training data were all correctly classified. Sulfamethoxazole and trimethoprim were mostly classified as control, in line with these drugs being individually inactive at the concentrations used (fig. S8). The two drugs with a previously unseen MoA (rifampicin and rifabutin) were split between two distinct “known” MoA classes: DNA gyrase and ribosome inhibition, consistent with our finding in Fig. 3B that our DL model clusters drugs targeting these related biological functions. In contrast, the previously trained random forest classifier was only able to correctly classify the two DNA gyrase inhibitors (BDM71403 and gepotidacin; fig. S5B).

Next, we computed outlier scores on feature vectors of candidate drugs using a local outlier factor algorithm, an approach that we developed in prior work for detecting drugs with novel MoAs in *E. coli* (Materials and Methods) (*32*). As expected, both rifampicin and rifabutin produced the highest outlier scores (0.96 and 0.80, respectively, compared to outlier scores ranging from 0.18 for ampicillin to 0.64 for BDM71403; fig. S9B). We then systematically explored the ability of our DL model to detect drugs with novel MoAs. For this, we retrained our DL model by leaving out all drugs belonging to a single MoA (as well as sulfamethoxazole and trimethoprim which served as negative controls) and computed outlier scores, as well as pairwise distances between drugs belonging to the left-out MoA which we compared to a null distribution of pairwise distances from randomly chosen and equal-sized sets of drugs to obtain a clustering score (Materials and Methods). We performed this analysis on six rotations of training and testing plates for drugs targeting PBPs, DNA gyrase, RNA polymerase, and ribosome. We found that median outlier scores were consistently higher for drugs with a previously unseen MoA compared to negative controls (fig. S10), and median clustering scores were consistently above the chance level for all considered MoAs (PBPs: 0.93, DNA gyrase: 0.98, RNA polymerase: 0.91, ribosome: 0.72; fig. S11). These results indicate that our DL model reliably extracts feature vectors for drugs with novel MoAs that are both distinct from other drugs and cluster together.

Overall, these results show that our DL model generalizes to previously unseen drugs and correctly matches active drugs to their correct MoA when other drugs with the same MoA are present in the training data and classifies inactive drugs as control. Images of bacteria exposed to drugs with a previously unseen MoA cluster together and apart from all other conditions which could pave the way for automated detection of novel classes of inhibitors directly from images. Our results also show that phenotypes of compounds targeting similar pathways can be grouped into functionally meaningful clusters from images alone.

### Linking drugs to mutants from high-throughput images

**Fig. 4.**
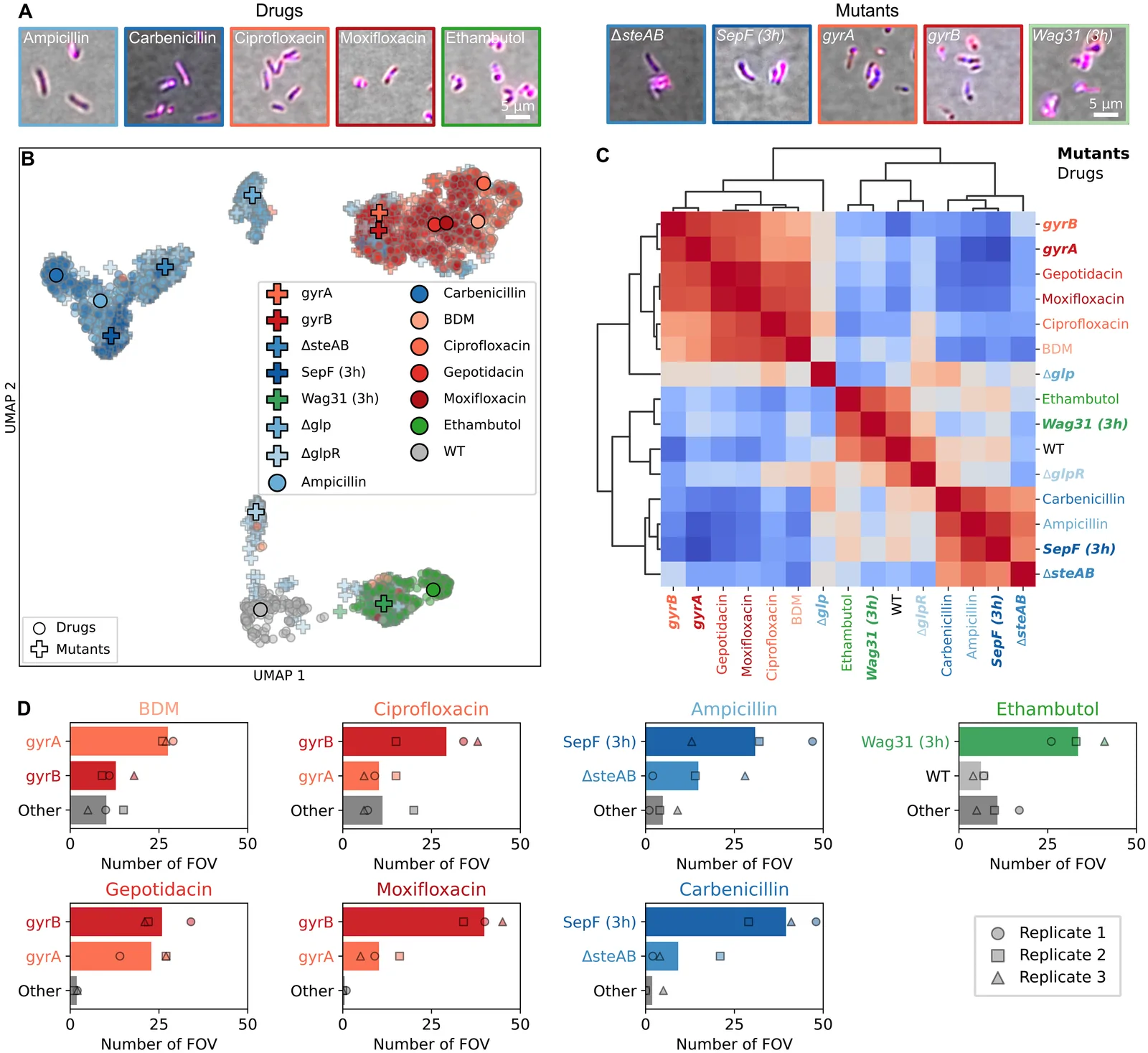
DL links drugs to mutants from images alone. (**A**) Image crops of drug-treated and mutant bacteria with scale bars. (**B**) UMAP of feature vectors colored by condition with small dots corresponding to images of bacteria treated with antibiotics at 10× MIC and crosses to mutants. Large dots/crosses correspond to median feature vectors computed across three biological and technical replicates. (**C**) Hierarchical clustering on a cosine similarity matrix of median feature vectors shows mutants and bacteria treated with antibiotics disrupting similar pathways clustering. (**D**) MLPs were trained to predict affected pathways from feature vectors of mutants using two of the three available plates (biological and technical replicates). Predictions on images of drug-treated bacteria were then obtained using a third hold-out test plate. In total, three models were trained and evaluated on three hold-out test plates (Materials and Methods).

If a drug acts on a single gene product or biological pathway, then a mutant strain genetically disrupted for the same gene or a gene involved in the same pathway should lead to a phenotype recognized as similar by our DL model. On the basis of this hypothesis, we asked whether we could identify the MoA of compounds by comparing drug-induced phenotypes to phenotypes of mutants in which the genes encoding the direct targets of the drugs or belonging to the same biological pathway/machinery are disrupted. As a proof of concept, we chose mutants affecting four major biological pathways of the polar growing *Cglu* (Table 2 and table S1): (i) strains enabling inducible repression of the genes *gyrA* and *gyrB* (*35*), which respectively encode the two subunits GyrA and GyrB of the essential DNA gyrase complex (*36*) that modifies DNA topology and is crucial for DNA replication and segregation; (ii) a strain for inducible repression of *sepF*, the gene coding for the membrane anchor of the tubulin homolog FtsZ (*37*) that is essential for Z-ring assembly and required for septum formation and cell wall biosynthesis; (iii) three different deletion mutants [Δ*steAB* (*38*), Δ*glp* (*39*), and Δ*glpR* (*39*)] of genes involved in the divisome-elongasome transition at the septum; and (iv) a conditional mutant *wag31* (*40*) encoding for the essential polar elongasome scaffold Wag31, which leads to disrupted polar cell wall biosynthesis. In addition to these four groups of mutants, we also included *Cglu* bacteria exposed to seven drugs at 10× MIC which target similar pathways (Table 2). We then prepared three plates with randomized layouts (Materials and Methods) containing the above-described mutants and drug-treated bacteria and imaged them as previously detailed (Fig. 4A).

First, we extracted feature vectors from the images of mutants and drug-treated bacteria in this new dataset, using our previous DL model without retraining, and plotted them with UMAP (Fig. 4B). Feature vectors obtained from the three replicates were in good agreement for all conditions, showing that our DL model was robust to changes in well positions and plate-to-plate variations (fig. S12A). We additionally computed a cosine similarity matrix on median feature vectors for all conditions and performed hierarchical clustering (Fig. 4C and Materials and Methods). Our analysis revealed three mixed clusters of drugs and mutants disrupting similar pathways. First, the four anti-gyrase drugs (BDM71403, ciprofloxacin, gepotidacin, and moxifloxacin) and the two DNA gyrase mutants (*gyrA* and *gyrB*) formed a tight cluster (Fig. 4, B and C). This is expected, since these drugs directly target the protein underlying the gyrase-depletion mutant. Unexpectedly, drugs interfering with cell wall biosynthesis (the PBP inhibitors ampicillin, and carbenicillin) clustered with two cell division mutants (*sepF* and Δ*steAB*). While the proteins produced by these genes are not the direct drug targets of these drugs, they are nonetheless directly linked to PG biosynthesis. For instance, ampicillin and carbenicillin clustered with an early time point *sepF* depletion mutant, as well as the Δ*steAB* deletion strain. SepF is the membrane anchor of the tubulin homolog FtsZ and thus essential for septum formation through a ring-like structure (Z-ring) that positions the PG biosynthetic machinery at the division site (*37*), while the SteAB complex regulates RipA, the major endopeptidase responsible for PG remodeling at cytokinesis (*38*). Hence, these two mutants, although not being depleted for PBPs (the direct targets of ampicillin and carbenicillin), are involved in regulating PG synthesis at the septum and form a physiologically relevant cluster with beta-lactam antibiotics. The AG inhibitor ethambutol, known to disrupt the ability of cells to elongate from their poles (*24*, *41*), consistently clustered with the *wag31* mutant which lacks the elongasome scaffold Wag31, essential for rod shape and pole maintenance.

The relative consistency between feature vectors of mutants and drugs affecting related pathways encouraged us to ask whether these features can be used to associate a mutated gene to each compound affecting this gene’s pathway. For this purpose, we trained a multilayer perceptron (MLP) with a single hidden layer using the DL-derived feature vectors obtained from images of mutants as input and the gene corresponding to the disrupted pathway as label. We then used the DL-derived feature vectors obtained from images of drug-treated bacteria to infer the disrupted pathways with the MLP trained on mutants across the three replicates (Fig. 4D and Materials and Methods). Images of bacteria exposed to the four DNA gyrase inhibitors BDM71403, ciprofloxacin, gepotidacin, and moxifloxacin were correctly associated to either *gyrA* or *gyrB* depletion mutants. The two PBP inhibitors ampicillin and carbenicillin were in turn associated to *sepF or* Δ*steAB*, mutants which are known to perturb the division and thus the septal cell wall machineries. Ethambutol was correctly associated to the cell elongation defective mutant *wag31*. In contrast, when using segmentation-based features to train a random forest classifier to predict disrupted genes, only ethambutol and ampicillin were correctly classified across all three hold-out test plates (fig. S13A), and even when training the classifier on broad pathways by grouping mutants into four classes (controls, DNA gyrase, division, and elongation), only four (gepotidacin, ampicillin, carbenicillin, and ethambutol) of the seven antibiotics were correctly classified across all three replicates (fig. S13B).

In summary, these results show that our DL model links mutants to drugs that perturb similar pathways directly from high-throughput images. The possibility of identifying the biological pathway disrupted by a drug from images alone represents an important step toward mutant-based target deconvolution of hit compounds by comparing drug-induced phenotypes to those found in genome-wide mutant libraries.

### Detection of mechanistic fingerprints in cell cycle–associated pathways

**Fig. 5.**
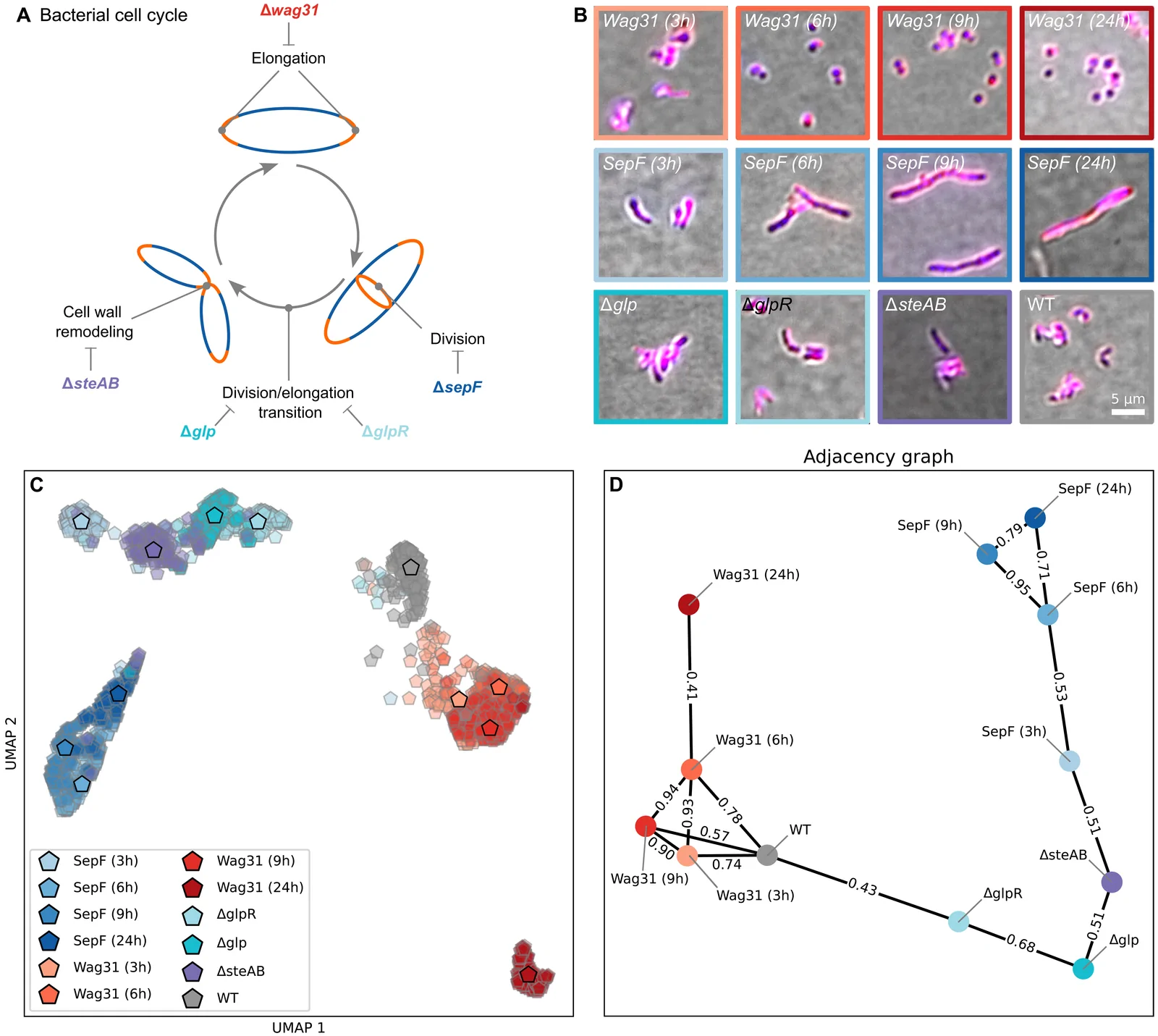
Images of bacterial cell cycle mutants cluster by disrupted pathway. (**A**) Diagram showing the bacterial cell cycle and pathways affected by mutants. (**B**) Representative image crops shown for each mutant with scale bars. (**C**) UMAP of image embeddings colored by mutants computed on three technical replicates with small pentagons corresponding to individual images and large pentagons to median feature vectors. (**D**) Adjacency graph obtained from a cosine similarity matrix (fig. S12C) computed on median feature vectors and colored by mutants showing connections between mutants with the most similar feature vectors. Values on edges correspond to cosine similarities between mutants. The threshold for the adjacency matrix was set such that each node was connected by at least one edge.

On the basis of the results above, wherein our DL model successfully linked phenotypes of drug-treated bacteria to mutants of the targeted pathways, we asked whether a similar approach could be used to provide insights into fundamental biological processes. As a proof of principle, we focused on five of the above-mentioned mutants implicated in the bacterial cell cycle and for which functional data are available in the literature (Fig. 5A and fig. S14). These included the three knockout strains Δ*steAB*, Δ*glp*, and Δ*glpR* and the two conditional depletion mutants *sepF* and *wag31*, which were imaged at four time points (3, 6, 9, and 24 hours) corresponding to increasing depletion levels. During Wag31 depletion, cellular morphologies evolved from rod to ovoid and lastly to coccoid shape (Fig. 5B) (*40*). Upon SepF depletion, the Z-ring disassembles, and cells start to elongate from roughly 2 μm in WT to more than 10 μm during depletion (*37*). These elongated cells eventually branch (Fig. 5B)—which corresponds to uncontrolled and aberrant pole formation—and lastly lyse. Glp and GlpR form a tight complex at the septum and respectively bind FtsZ and Wag31, thus linking the division and elongation cytoskeleta at the septum to ensure the correct transition between division and elongation. Glp depletion results in elongated cells, while GlpR depletion resembles WT cells in classical morphological readouts (Fig. 5B and fig. S14) (*39*). Last, SteAB forms a complex involved in cell wall remodeling by regulating PG hydrolysis and is also responsible for correct onset of cytokinesis (*42*, *43*). Δ*steAB* cells are elongated and usually display multiple septa (Fig. 5B and fig. S14) (*42*, *44*).

We imaged the above-mentioned mutants in triplicates as previously described (Materials and Methods) and used our DL model trained on antibiotics (Table 1) to obtain feature vectors which showed low plate-to-plate variation across replicates (fig. S12B). Plotting the extracted feature vectors with UMAP revealed clustering of mutants consistent with different stages of the bacterial cell cycle (Fig. 5C). Early time point mutants (3, 6, and 9 hours) of Wag31 formed a tight cluster and were the closest to the WT cells compared to all other mutants. This is consistent with the fact that early depletion of *wag31* leads to a relatively subtle but significant phenotype of cellular rounding (*40*). The late time point mutant of *wag31* (24 hours) localized away from all other conditions reflecting its distinctly rounded shape. All *sepF* mutants, except for the earliest time point at 3 hours, formed a cluster that was the furthest away from WT cells, reflecting the extreme elongation phenotype induced by interfering with FtsZ function (Fig. 5B) (*37*). The early time point sepF mutant was near the three knockout mutants Δ*steAB*, Δ*glp*, and Δ*glpR*, known to be perturbed in their division machinery. The Δ*glpR* mutant, which did not differ significantly from WT cells when analyzed using segmentation and classical morphological features (*39*), clustered away from WT and together with the Δ*glp* mutant. Glp and GlpR are known partners in vivo where they localize to the septum during cell division (fig. S14). They have also been shown to form a strong complex in vitro (nanomolar range affinities) (*39*). This suggests that the DL model can pick up relevant physiological signatures overlooked by classical cell analytical methods.

To further quantify these observations, we constructed an adjacency graph (Fig. 5D) from a cosine similarity matrix computed on median feature vectors (fig. S12C) setting the threshold such that each node was linked by at least one edge (Materials and Methods). Mutants disrupting cell elongation and cell division occupied the extremes with higher depletion levels being the furthest away from all other conditions. Mutants associated with the division machinery were localized between these two extremes. Notably, our adjacency graph recovers the known function of these proteins in the bacterial cell cycle, as shown in Fig. 5A and fig. S14. For example, Δ*steAB* and Δ*glp* both lead to multiseptated elongated phenotypes that prevent correct cell cycle progression (*39*, *42*), and Δ*glpR* falls in between Δ*glp* and WT. Next, we constructed an adjacency graph using hand-crafted feature vectors (fig. S15). However, while *wag31* and *sepF* correctly localized at the extremes, different depletion levels were not separated, and the three mutants Δ*steAB*, Δ*glp*, and Δ*glpR* were not clearly distinguished with multiple *sepF* mutants at different depletion levels connecting to Δ*steAB.*

Together, these results show that the images of mutant strains carry valuable information for understanding gene function. Our DL model recovers known mechanistic relationships between bacterial genes, thus paving the way toward genome-wide studies of bacterial gene function directly from imaging data.

## DISCUSSION

In this work, we present a DL-based analysis approach to extracting highly specific features from high-throughput images of chemically and genetically perturbed *Cglu* bacteria. We show that these features enable robust discrimination between drugs by MoA and recognition of the MoA of previously unseen drugs. We also demonstrate that drugs with a previously unseen MoA score as outliers and cluster in feature space, suggesting a path toward image-based detection of drugs with novel MoAs. Moreover, we demonstrate that our approach leads to significantly higher classification accuracies compared to a random forest classifier trained on hand-crafted features. In addition, we find that our DL model correctly classifies all but one drug with a previously seen MoA at a concentration above 5× MIC. Then, we demonstrate that the learned features allow to link MoA-specific chemical perturbations to genetic perturbations belonging to the same biological pathway for three relevant processes commonly targeted by antibacterial drugs: DNA gyrase, elongation, and division/cell wall machineries. Last, we show encouraging results for annotating bacterial gene function directly from images using the cell cycle of *Cglu* as a case study.

We used *Cglu* as a surrogate model to develop a pipeline to screen for TB inhibitors. Ideally, screens should be carried out using the pathogen in question. However, in this regard, not all bacteria are equal. While traditional bacterial cytological profiling (BCP) is sufficient to determine the MoA in *E. coli* or *Bacillus subtilis*, this is not the case for *Mtb* (*13*, *14*, *16*, *17*). BCP performs poorly on mycobacteria (*16*, *17*), and DL methods have only recently been used to successfully distinguish drug-induced phenotypes in *Mtb* (*17*)*.* A key obstacle of using classical methods to profile *Mtb* is the intrinsic cell-to-cell variability of the tubercle bacillus (*45*). Elegant work by Smith *et al.* (*16*) demonstrated that standard phenotypic analysis is not sufficient to distinguish drug-induced phenotypes of *Mycobacteria* precisely because of this cellular heterogeneity as well as subtle morphological responses to drugs that tend to be masked by the cell-to-cell variability. To address this, the authors developed a complex pipeline (MorphEUS) that considers cellular heterogeneity and relies on iterative feature selection including variables linked to cell-to-cell variation. Although their pipeline can address detailed questions around known MoAs and off-target effects of a small number of compounds, it would be challenging for high-throughput screening of large compound libraries. Moreover, while these methods enable MoA recognition of hit compounds with respect to known compounds, they are unable to identify drug targets of compounds that exhibit novel MoAs. Our results suggest that we can identify novel/unseen MoAs, and we address the above limitation by linking drugs to mutants where similar pathways have been disrupted directly from high-throughput images (Fig. 4).

Another potential limitation of these prior studies is the choice of model organism. To overcome the limitations of working on *Mtb* directly while keeping a parent model that retains core conserved cellular pathways, we chose the industrially relevant bacterium *Cglu*. It is not pathogenic, substantially simplifying the experimental pipeline, and grows rapidly (doubling time 1 hour) in defined media. This is a crucial consideration when producing data for DL-based analysis where reproducible phenotypic readouts and minimal batch-to-batch variation are key, as even slight variations in media composition can lead to important morphological alterations (*46*). In addition, drug-induced phenotypes are more homogeneous when compared to *Mtb*, thereby simplifying the analysis. We therefore believe that *Cglu* is an attractive model organism to screen for novel TB drugs. In particular, the similar and unique cell wall composition [a major target for anti-TB treatment (*47*)] and the conserved core machinery across the two species are important advantages. We do, however, acknowledge that relevant TB targets in this surrogate model will be limited to conserved mechanisms and that this model will not be suited for finding *Mtb-*specific pathways such as those linked, for example, to virulence or host adaptations.

In prior work, we showed that brightfield images alone are sufficient to distinguish between drug MoAs in *E. coli* bacteria (*32*). Despite the absence of clearly discernible visual phenotypes in most conditions (Fig. 2A), our DL model produced good results on the dataset of *Cglu* bacteria only using the brightfield channel (fig. S16A). However, the addition of fluorescent dyes significantly (*P* = 0.031, Wilcoxon signed-rank test) improved prediction accuracies (79.16 ± 7.25% versus 87.25 ± 7.27% computed per FOV; fig. S16B). This is likely due to drug-treated *Cglu* bacteria exhibiting much subtler phenotypes compared to *E. coli*.

In the context of a phenotypic screen, early identification of the hit’s MoA is crucial to select promising compounds for further downstream development. Since our approach can recognize the MoA of previously unseen compounds directly from images by comparison to drugs with known MoAs or detect that they have a novel MoA (Fig. 3 and fig. S10), it sidesteps laborious experiments in the laboratory. By providing insights on the MoA directly from imaging data generated in a screen, our method is a step toward rational hit selection. Our data also suggest that compounds with previously undiscovered MoAs could quickly be detected, and their target pathways identified by comparing their phenotypes to those of mutants.

We also observed that predictions from our DL model were correct for all but one drug above 5× MIC, and for a subset of drugs, MoA predictions were correct regardless of concentration (fig. S7A). Notably, if these drugs had been used in the context of a screen at 10 μM, then the MoA of only 2 (clofazimine and ethambutol) of the 14 active drugs with a previously seen MoA would not have been correctly identified. While this is encouraging, it should be noted that these drugs have undergone extensive lead optimization and are thus more potent than typical hit compounds. It therefore remains to be explored how DL performs on large nonspecific compound libraries.

Another direct application of this work is the possibility for an immediate validation of compound series resulting from medicinal chemistry. Compounds can be rapidly tested for both cell penetrability and efficacy on the target, as well as possible off-target effects resulting from chemical modifications. Since our DL model encodes dose-dependent phenotypic changes of drug-treated bacteria on MoA-specific trajectories in the feature space (fig. S7, B and C), we speculate that our approach might also be used to aid lead optimization of hit compounds through image-based structure activity relationship (SAR) studies. SAR landscapes could be constructed on the basis of the features extracted by our DL model from images of drug-treated bacteria to map the potency of a given change in molecular structure onto these dose-dependent trajectories. This could enable high-throughput studies where the molecular structure of a lead compound is systematically altered to gain insights on potency directly from images.

Making a direct link between mutants and drugs is only possible in very few well documented cases such as DNA gyrase inhibitors for which the target is possibly unique and experimental structures of drug-target complexes are available (*48*). In contrast, in the case of PBPs, one drug may target several enzymes, and mutant strains will not reflect a one-target-one-drug scenario. Other drugs used in the clinic have controversial target attributions, and many MoAs cover global physiological perturbations, as exemplified by clofazimine, which at least in part targets the respiratory chain but has also been reported to have more general effects on disrupting membrane potential and homeostasis (*30*). Dissecting these possible effects will be of great importance, not only for MoA detection but also for quantifying off-target effects that could be more general and lead to toxicity. While our results (Fig. 4) remain to be validated on larger datasets, we believe that they point toward a strategy of target deconvolution by relating drug-induced phenotypes to whole-genome mutant libraries.

Our results have also shown that we can not only detect the markedly different phenotypes linked to a complete disruption of cell division (SepF) or elongation (Wag31) but also more subtle phenotypes that cannot be distinguished in classical segmentation-based feature analysis (Fig. 5). One such example is the GlpR mutant strain that has a WT morphology when segmented and analyzed for cell parameters such as length and width (*39*). Our analysis, however, reveals clear differences to the WT condition (Fig. 5C) and high similarity to the Glp strain, in line with the known biological function of GlpR. GlpR has been shown to form a strong complex with Glp (nanomolar range) at the corynebacterial septum during cell division (*39*). This shows that our DL model picks up functionally relevant features that escaped classical analysis.

In conclusion, we have demonstrated that images of bacteria contain a high degree of pathway-specific phenotypic features and have introduced a DL approach capable of extracting this rich information source. With growing datasets facilitated by automated screening protocols, methods like the one presented herein are poised to greatly speed up the discovery of novel antibiotics and chart a path toward systematically investigating bacterial gene function directly from imaging data.

## MATERIALS AND METHODS

### Bacterial strains, culture conditions, and antibiotic treatment

*C. glutamicum* American Type Culture Collection (ATCC) 13032 (*Cglu*) was used as a WT strain. *Cglu* was grown overnight in a flask in CGXII medium (*49*) with 4% sucrose at 30°C and 110 rpm shaking. The following day, the culture was diluted to OD_600_ of 1.3 in a flask in CGXII medium with 4% sucrose, grown for 2.5 hours at 30°C and 110 rpm (to a required OD_600_ of 2), and then dispatched in a 96-well plate containing randomized antibiotics. Cells were then treated for 6 or 16 hours at 30°C and 110 rpm, before being fixed as described below. *Cglu_P_ino_-wag31* and *Cglu_P_ino_-sepF* strains were inoculated in CGXII 4% sucrose and grown for 8 hours at 30°C 110 rpm and then diluted to OD_600_ of 1.3 in CGXII 4% sucrose supplemented with 1% myo-inositol and grown for either 3, 6, 9, or 18 hours at 30°C 110 rpm for different depletion levels. *Cglu_P_ino_-GA* and *Cglu_P_ino_-GB* were inoculated in CGXII 4% sucrose and grown overnight at 30°C 110 rpm, diluted the next day to OD_600_ of 1.3 in CGXII 4% sucrose 1% myo-inositol, and grown at 30°C 110 rpm for 7 hours and 30 min. Cultures were then diluted again to an OD_600_ of 1.3 in fresh CGXII medium with 4% sucrose 1% myo-inositol and grown for 18 hours at 30°C 110 rpm to reach total depletion. *Cglu_*Δ*glp*, *Cglu_*Δ*glpR*, and *Cglu_*Δ*steAB* were inoculated in CGXII 4% sucrose, grown overnight at 30°C 110 rpm, diluted the next day to OD_600_ of 1.3 in CGXII 4% sucrose, and grown for 5 hours at 30°C 110 rpm.

### Antibiotic dispatching

For the dataset used for training, antibiotics were dispensed by acoustic droplet ejection using an Echo 550 liquid handler (Beckman Coulter Life Sciences) into dry 96-well plates (clear, flat bottom, TPP 92097). Well positions for each assay plate were randomized (fig. S1) before the Echo run; randomized pick lists were generated upstream and imported into Echo Cherry Pick to create the transfer instruction files. Compounds from the Food and Drug Administration–approved and Passed Phase I Drug Library (Selleckchem L3800) included amoxicillin, ampicillin, cefotaxime, doxycycline, moxifloxacin, rifabutin, rifampicin, linezolid, carbenicillin, clarithromycin, trimethoprim, clofazimine, ethambutol, sulfamethoxazole, and ciprofloxacin. Additional compounds were gepotidacin (MCE, HY-16742), novobiocin (Sigma-Aldrich, N1628), and BDM71403 (*23*). All antibiotics were dispensed from DMSO stocks except novobiocin which was dispensed from aqueous stocks. For each antibiotic, four concentrations corresponding to 0.5, 1, 5, and 10× MIC were dispensed. Wells containing DMSO were backfilled to achieve a uniform final DMSO content of 0.49% in the assay. Control wells included six vehicle controls with 0.49% DMSO and six vehicle controls with water. Immediately after acoustic transfer, 10 μl of growth medium were added to each well to minimize evaporation and compound degradation, followed by culture addition. The second dataset was generated for a subset of antibiotics (Table 1) as described above, with the addition of higher concentrations (50 and 100× MIC). We note that ciprofloxacin was only compatible up to a concentration of 50× MIC.

For the third dataset (Fig. 4), all dilutions were made through manual pipetting. Moxifloxacin (Sigma-Aldrich, SML1581), BDM71403 (*23*), and gepotidacin (MCE, HY-16742) were diluted in DMSO and added to the culture at a final DMSO concentration of 0.1%. Ciprofloxacin (Sigma-Aldrich, PHR1044), ethambutol (Sigma-Aldrich, 1070-11-7), carbenicillin (Euromedex, 1039A), and ampicillin (Sigma-Aldrich, A9518) were diluted in water. Drugs were dispensed at a concentration of 10× MIC values listed in Table 1.

### MIC determination

The MIC determination against WT *Cglu* ATCC13032 was determined by the agar dilution method on Mueller-Hinton (MH) agar and broth. For the agar dilution method, MIC values were obtained using the previously described proportion method (*50*). Briefly, 10^3^ and 10^5^ colony-forming units were inoculated onto MH agar containing twofold serial dilutions of each compound. After 1 day of incubation at 30°C, colonies were enumerated. The MIC was defined as the lowest concentration of antibiotic resulting in complete inhibition of growth or in growth of fewer than 1% of the inoculum. For the broth dilution method, antibiotics were prepared in twofold serial dilutions in a 96-well flat-bottom plate, each well containing 100 μl of MH broth with the corresponding antibiotic. Wells were inoculated with 2 μl of an overnight culture. Plates were incubated for 24 hours at 30°C with 600 rpm shaking. OD_600_ values were measured at 24 hours. MIC was determined as the absence of growth compared to blank.

### Sample fixation and preparation for imaging

For 96-well plates, 75 μl of bacterial cultures was mixed with 180 μl of ice-cold ethanol (EtOH) and stored for 1 hour at −20°C. Samples were washed three times with 500, 700, and 75 μl of filtered sterile phosphate-buffered saline (PBS) and diluted to OD_600_ of 0.025 in PBS supplemented with FM4-64 (1 μg/ml final; Invitrogen, T13320) and Hoechst 33342 (1 μg/ml final; Cell Signaling Technology #4082). One hundred microliters of each sample was loaded in a PhenoPlate (Revvity, 6055302), which was stored at 4°C and centrifuged before imaging. Those steps were either done manually either using an Apricot PP5 robot (sptlabtech). For mutant samples, 300 μl of each culture was harvested and fixed with 700 μl of ice-cold EtOH for 1 hour at −20°C. Samples were then washed three times in 300 μl of filtered sterile PBS and diluted to OD_600_ of 0.025 in PBS supplemented with FM4-64 (1 μg/ml final, Invitrogen T13320) and Hoechst 33342 (1 μg/ml final; Cell Signaling Technology #4082). One hundred microliters of each sample was loaded in a PhenoPlate (Revvity, 6055302) following a random map. Plates were stored at 4°C and centrifuged before imaging.

### High-throughput imaging

Plates that were stored at 4°C were left at least 30 min at room temperature and centrifuged for 10 min at 500 rpm. An Opera Phenix Plus (Revvity) was used in widefield mode to image 50 FOVs per well, with a 63/1.15 water-immersion objective. The fluorescent dyes—Hoechst 33342 and FM4-64—were acquired simultaneously with 405- or 488-nm excitation laser lines and 435- to 480-nm or 650- to 680-nm bandpass filters, respectively, in addition to brightfield imaging. The XY image resolution was 0.0941867 μm.

### Image preprocessing

Images were converted from 16 bit to 8 bit, and pixel intensities were clipped by percentile between 0.1 and 99.9 to remove spurious saturated pixels. Images from five of six plates were randomly split into training (80%) and validation (20%) sets. Data augmentation was applied to full-resolution images (2160 × 2160 pixels) on the fly (random flipping and rotation, random changes in brightness, contrast, saturation, and hue). Randomly rotated images were then center-cropped (1528 × 1528 pixels) to account for potential edge effects. Next, a random crop (1500 × 1500 pixels) was obtained and divided into nine tiles (500 × 500 pixels) which were resized to 256 × 256 pixels, and pixel intensities were normalized between −1 and 1.

### CNN architecture and training

A custom CNN was implemented in PyTorch v2.3.1 based on an EfficientNet-B0 (*51*) backbone. The CNN was first pretrained for 100 epochs using an Adam (*52*) optimizer with a learning rate of 0.001 and an effective batch size of 144 images (16 images divided into 9 tiles). Image tiles were passed through the backbone, with weights initialized by pretraining on ImageNet, and latent embedding vectors corresponding to individual image tiles were aggregated by averaging to obtain a global embedding vector for each input image of size 1500 × 1500 pixels. For pretraining, the model was trained to predict the combination of drugs and concentrations as a multiclass classification task using a weighted cross-entropy loss (classes were inversely weighted by the number of images in the training data).

For fine-tuning, model weights corresponding to the lowest validation loss during pretraining were chosen, and only images corresponding to drug treatments at the highest concentration (10× MIC) were used for training. Images of bacteria exposed to drugs belonging to the same MoA were grouped into the same class. During fine-tuning, models were trained using a combination of three loss terms with latent embedding vectors obtained from an intermediate fully connected layer of 16 neurons. First, a triplet loss (*53*) was used to explicitly promote the formation of clusters by MoA in latent space, defined as

$$L_{\text{triplet}}=\sum_{i=1}^{m}\text{max}\left({\left\|a_{i},p_{i}\right\|}_{2}^{2}-{\left\|a_{i},n_{i}\right\|}_{2}^{2}+\alpha ,0\right)$$

where $a_{i}$ denotes the ith anchor embedding vector, $p_{i}$ denotes the ith embedding vector of a positive example, and $n_{i}$ denotes the ith embedding vector of a negative example, while m corresponds to the number of hard triplets. The margin α was set to 0.1. During training, triplets were assembled on the fly based on their MoA class. In addition, hard triplet mining was applied, such that the loss was only computed on triplets where the negative example was closer to the anchor than the positive example. Therefore, hard triplets satisfy the following inequality

$${\left\|a_{i},n_{i}\right\|}_{2}^{2}<{\left\|a_{i},p_{i}\right\|}_{2}^{2}$$

Second, a center loss (*54*) was used to increase the tightness of clusters. The center loss seeks to minimize intraclass variations while ensuring that different classes remain separable and is thus defined as

$$L_{\text{center}}=\frac{1}{2}\sum_{i=1}^{m}{\left\|x_{i}-c_{y_{i}}\right\|}_{2}^{2}$$

where $x_{i}$ denotes the ith embedding vector, $c_{y_{i}}$ denotes the center of the $y_{i}$th class corresponding to the MoA of a given embedding, and m is the effective batch size.

Third, a weighted cross-entropy loss was used to obtain MoA predictions (weighted by the inverse number of training images per class). The final loss function used for training was thus

$$L=L_{\text{triplet}}+0.5\cdot L_{\text{center}}+L_{\text{cross}–\text{entropy}}$$

### Hierarchical clustering

Hierarchical clustering was computed on 16-dimensional feature vectors with Scipy v1.12.0 using the “scipy.cluster.hierarchy” function with the Ward’s variance minimization algorithm (“ward”). The Ward’s method was selected since it is designed to minimize the increase in within-cluster variance at each agglomerative step thus leading to compact and internally homogeneous clusters. The clustering threshold was set with respect to the negative control conditions (water and DMSO).

Hierarchical clustering of cosine similarity matrices was performed using the “seaborn.clustermap” function in Seaborn v0.11.2, which relies on the scipy.cluster.hierarchy function to cluster the underlying 16-dimensional feature vectors. The distance between clusters was computed with the Farthest Point Algorithm (“complete”) since it is compatible with the cosine distance metric and tends to produce well-separated clusters, ensuring consistency with the cosine similarity matrix.

### Local outlier factor algorithm

Embedding vectors from images of previously seen conditions were obtained from the hold-out test plate. A local outlier factor classifier was applied to these embeddings with the scikit-learn package in Python 3.9. The number of *k* neighbors was set to *k* = 10, and cosine distance was used as the distance metric as described in (*32*). Outlier scores were computed by taking the fraction of detected outliers considering all FOVs for a given condition.

### Clustering score

The intra-MoA clustering score was calculated from the pairwise L2 distances between median embedding vectors of compounds belonging to the same MoA. The observed mean pairwise distance was compared to a null distribution obtained from *n* = 10,000 randomly sampled and equal-sized sets of compounds. The clustering score was defined as the proportion of values in the null distribution that were greater than or equal to the observed mean pairwise distance such that higher scores indicate tighter clustering.

### Multilayer perceptron

Embedding vectors of 16 dimensions were obtained from images of mutants across three replicates. The “sklearn.neural_network.MLPClassifier” class from scikit-learn v1.5.1 was then used to construct a MLP with a single hidden layer of six neurons using a ReLU nonlinearity. MLPs were trained for 200 iterations with Adam and a learning rate of 0.001 to predict affected pathways from 16-dimensional embedding vectors of mutants using two of the three available plates. Predictions on images of drug-treated bacteria were then obtained using a third hold-out test plate. In total, three MLP models were trained and evaluated on three hold-out test plates.

### Adjacency graph

A cosine similarity matrix was first computed on 16-dimensional median embedding vectors. Then, a binary mask was obtained by thresholding the cosine similarity matrix. The threshold was set such that each node was connected by at least one edge. The element-wise product between the mask and the cosine similarity matrix was then computed to obtain a masked cosine similarity matrix. To obtain an adjacency matrix, the diagonal of this masked cosine similarity matrix was set to 0. An adjacency graph was then constructed from the adjacency matrix with the networkx v3.2.1 package using a spring layout to visually convey the cosine similarity between nodes with higher cosine similarities resulting in nodes localizing closer to each other.

### Hand-crafted feature extraction

Images were analyzed using the SImA software (v1.5.1, Revvity). First, advanced flat-field and brightfield corrections were applied to correct illumination inhomogeneity, using the embedded tools in SImA. Then, the merged image from the DNA and membrane channels was used to segment bacteria, using the Find Spots building block (method D). Morphological parameters were extracted, and to remove segmentation artifacts, only bacteria with an area ≥0.5 μm^2^ were kept for further processing. Additional morphological (STAR method) and texture (SER method, with a scale of 1 px) features were then calculated on the basis of the segmentation mask of each bacterium, for each of the three individual channels (DNA, membrane, and brightfield). The median values per well were collected for further analysis.

### Random forest classifier

Random forest classifiers were trained using 528-dimensional hand-crafted feature vectors as input with *n* = 1000 trees. Models were implemented using scikit-learn v1.5.1 in Python 3.9 with the “sklearn.ensemble.RandomForestClassifier” class. Reported classification accuracies were obtained from independent hold-out test replicates.
