# Supplementary material for: Deep learning extracts MoA-specific signatures from high-throughput images of chemically and genetically perturbed Corynebacteria
Source: Sci Adv. 2026 Sep 25;12(39):eaeg6806. doi: 10.1126/sciadv.aeg6806 (PMC13614406; doi:10.1126/sciadv.aeg6806)
Supplement: Supplementary file 1 [file sciadv.aeg6806_sm.pdf]

Science Advances
AAAS

Supplementary Materials for

Deep learning extracts MoA-specific signatures from high-throughput images of chemically and genetically perturbed *Corynebacteria*

Daniel Krentzel *et al.*

Corresponding author: Daniel Krentzel, daniel.krentzel@pasteur.fr; Christophe Zimmer, christophe.zimmer@pasteur.fr; Anne Marie Wehenkel, anne-marie.wehenkel@pasteur.fr

Sci. Adv. **12**, eaeg6806 (2026)
DOI: 10.1126/sciadv.aeg6806

The PDF file includes:

Figs. S1 to S16
Table S1
Legend for data S1

Other Supplementary Material for this manuscript includes the following:

Data S1

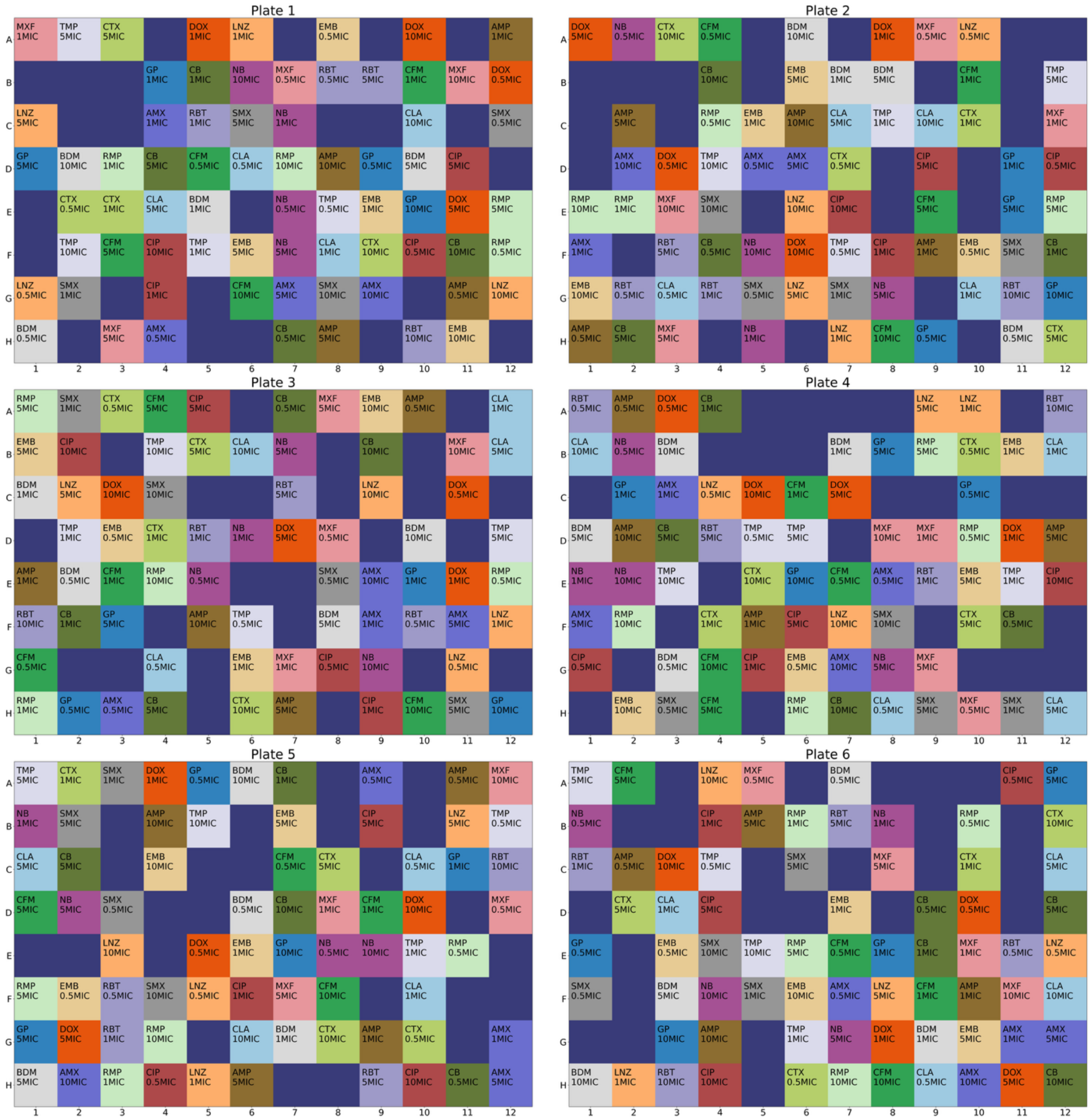

Figure S1. Plate layouts. Annotated plate layouts with drug concentration shown below. A total of 18 drugs at four different concentrations were distributed with an acoustic liquid handler. Amoxicillin: AMX, carbenicillin: CB, cefotaxime: CTX, ampicillin: AMP, ethambutol: EMB, ciprofloxacin: CIP, moxifloxacin: MXF, novobiocin: NB, gepotidacin: GP, BDM: BDM71403, clarithromycin: CLA, doxycycline: DOX, linezolid: LNZ, clofazimine: CFM, rifampicin: RMP, rifabutin: RBT, trimethoprim: TMP, sulfamethoxazole: SMX. Empty positions represent controls (water and DMSO).

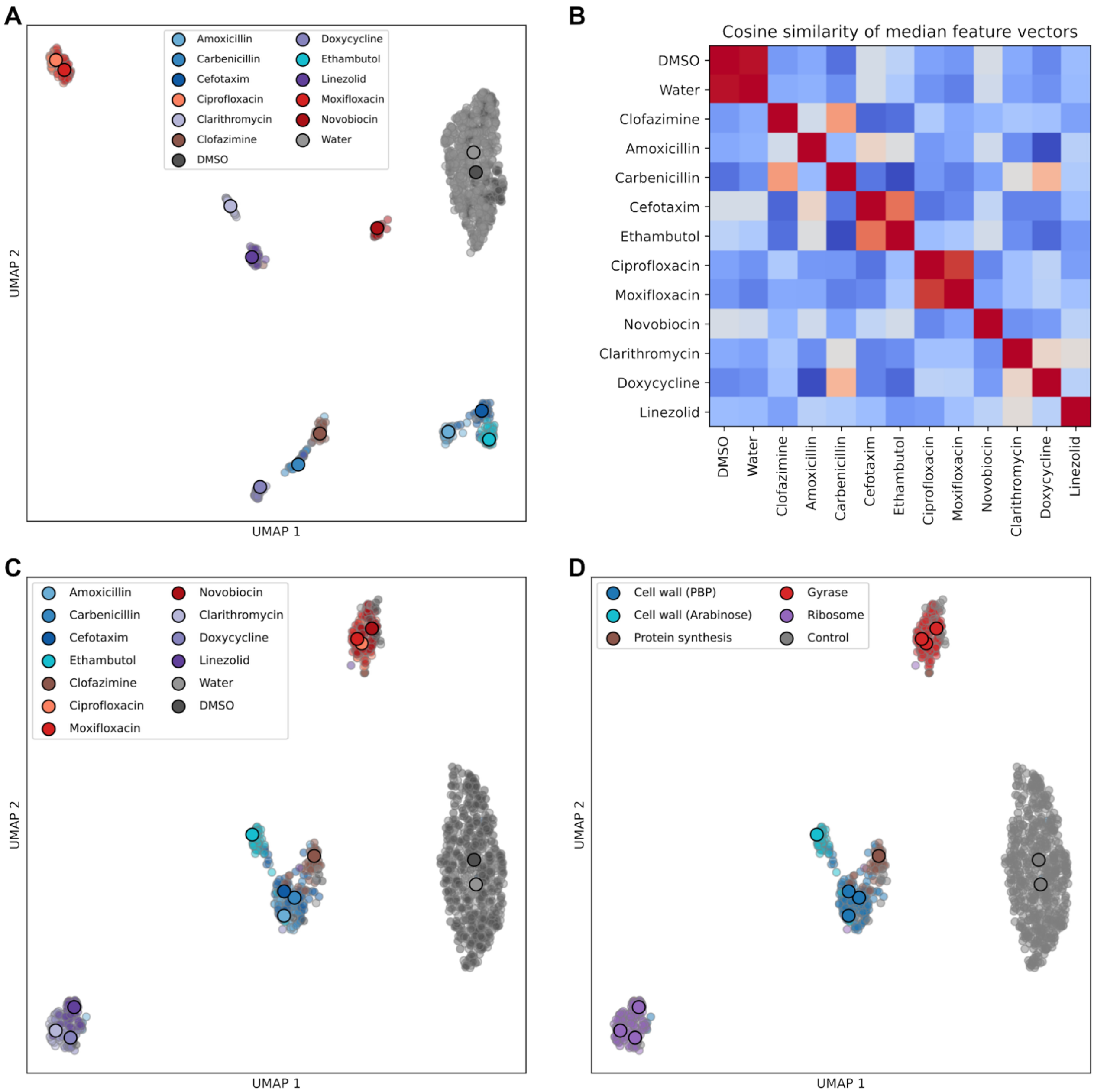

Figure S2. Comparison of feature vectors before and after fine-tuning. (**A**) UMAP plot of feature vectors obtained from images of drug-treated bacteria at 10xMIC from a model trained on the pretext task of classifying the combination of antibiotic and concentration without further fine-tuning (Materials and Methods). (**B**) The cosine similarity matrix was computed on median feature vectors that were obtained from images of drug-treated bacteria using a model that did not undergo fine-tuning. (**C**,**D**) UMAP plot of feature vectors using a model that was further fine-tuned to predict the mode of action (MoA) of antibiotics at 10xMIC (Materials and Methods) coloured by drug label (**C**) and MoA (**D**). Small dots correspond to individual fields-of-view (FOVs), and large dots correspond to the median feature vector of a given condition.

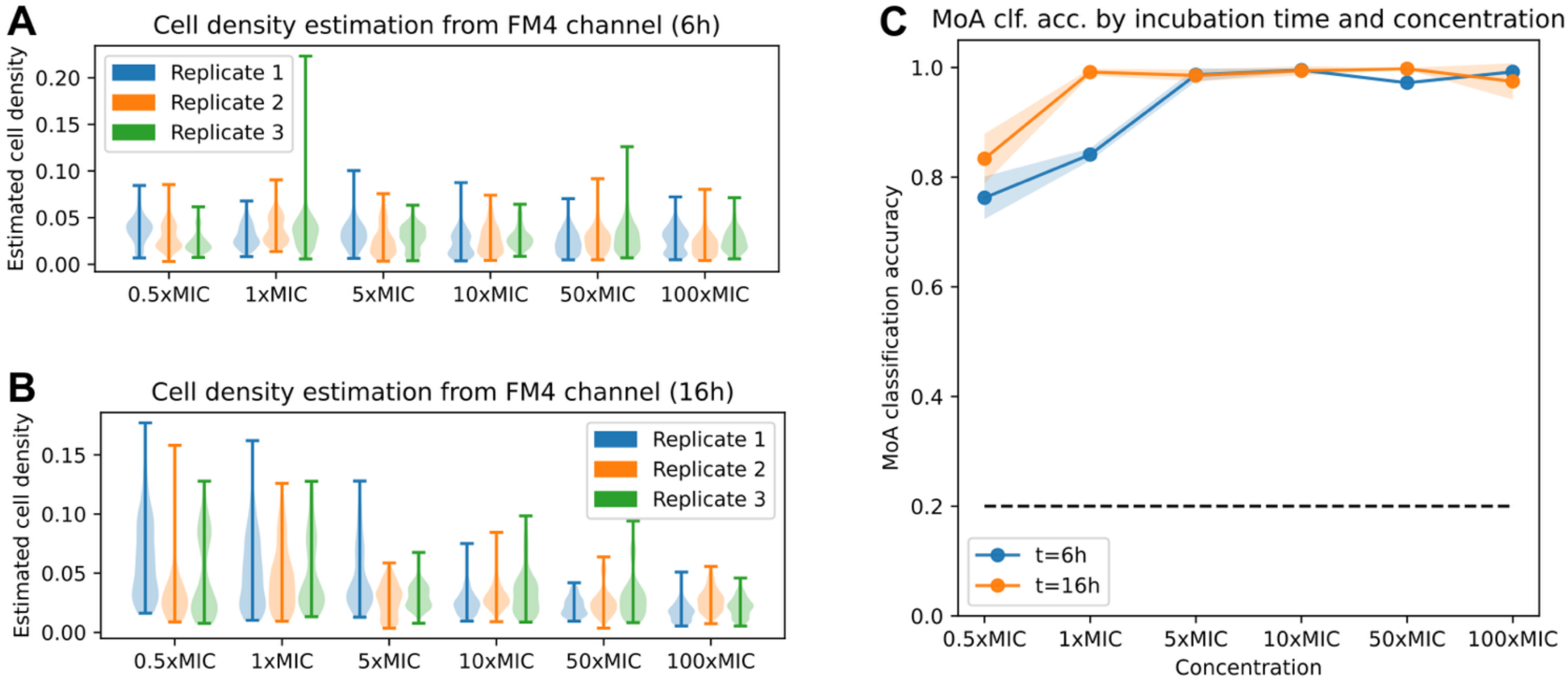

Figure S3. MoA classification accuracies for different incubation times and concentrations. (**A, B**) Cell densities were estimated by thresholding the FM4-64 channel using Otsu's method and then computing the fraction of the image area occupied by FM4-64 signal. Cell densities were estimated on all available images at each concentration and for each replicate at two time-points: 6 (**A**) and 16h (**B**). (**C**) DL models were fine-tuned on images at a single concentration and time-point using two training replicates and tested on images from the same condition (i.e. concentration and time-point) using a third hold-out test replicate in a cross-validation experiment. Coloured lines correspond to the mean accuracy across n=3 replicates and shaded areas indicates the standard deviation. The dashed line shows the random classification accuracy.

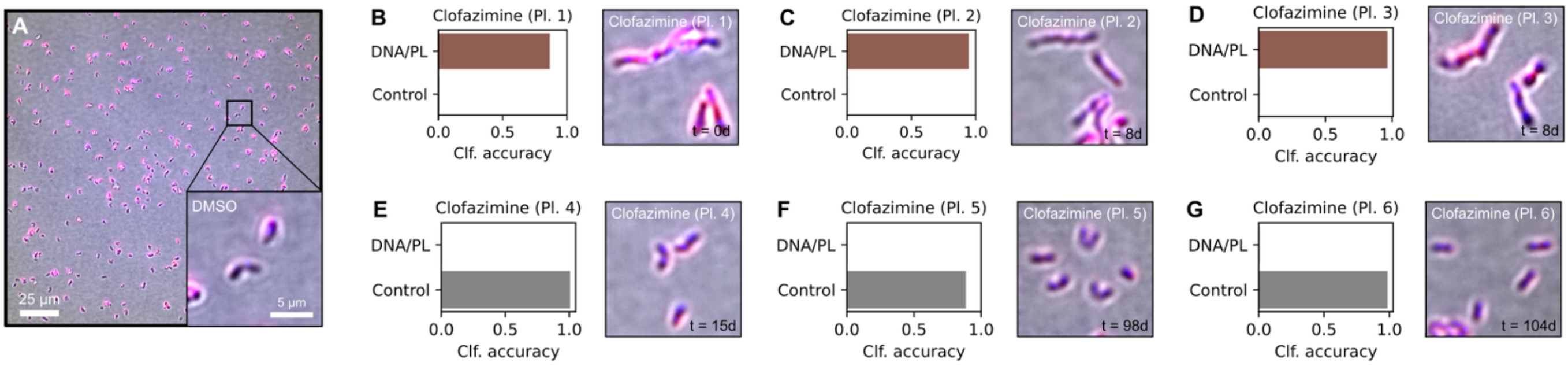

Figure S4. Visual inspection of clofazimine. (**A**) An example FOV of untreated cells (DMSO) with inset shown. (**B-G**) Per-plate classification accuracies for clofazimine (left) and example images for each corresponding plate with the number of days after the initial plate acquisition at which each plate was acquired shown in the bottom right corner (right). Images are of the same size as the inset in (**A**).

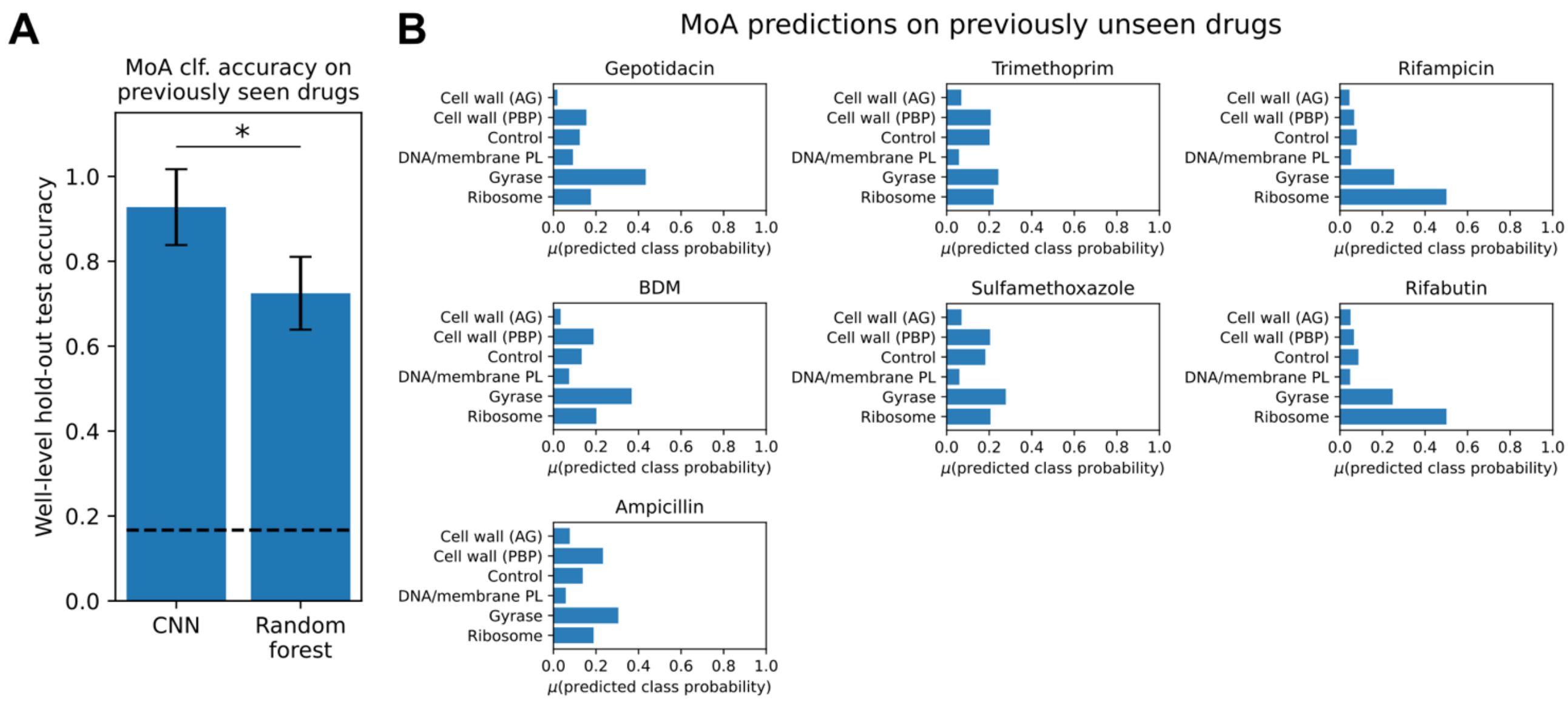

Figure S5. Benchmarking CNN against a random forest classifier trained on hand-crafted features. (**A**) Well-level MoA classification accuracies obtained with a DL model ('CNN') and a random forest classifier trained on 528 segmentation-based hand-crafted features ('Random forest') cross-validated across six hold-out test plates. Bars show mean accuracies with error bars indicating the standard deviation. MoA classification accuracies are 92.75±8.93% for the DL model and 72.46±8.57% for the random forest classifier. The dashed line shows the random accuracy. Statistical significance was computed with a two-sided Wilcoxon signed-rank test (p=0.031) for n=6 plates. (**B**) MoA predictions on previously unseen drugs using a random forest classifier. See Figure S9A for predictions using a DL model.

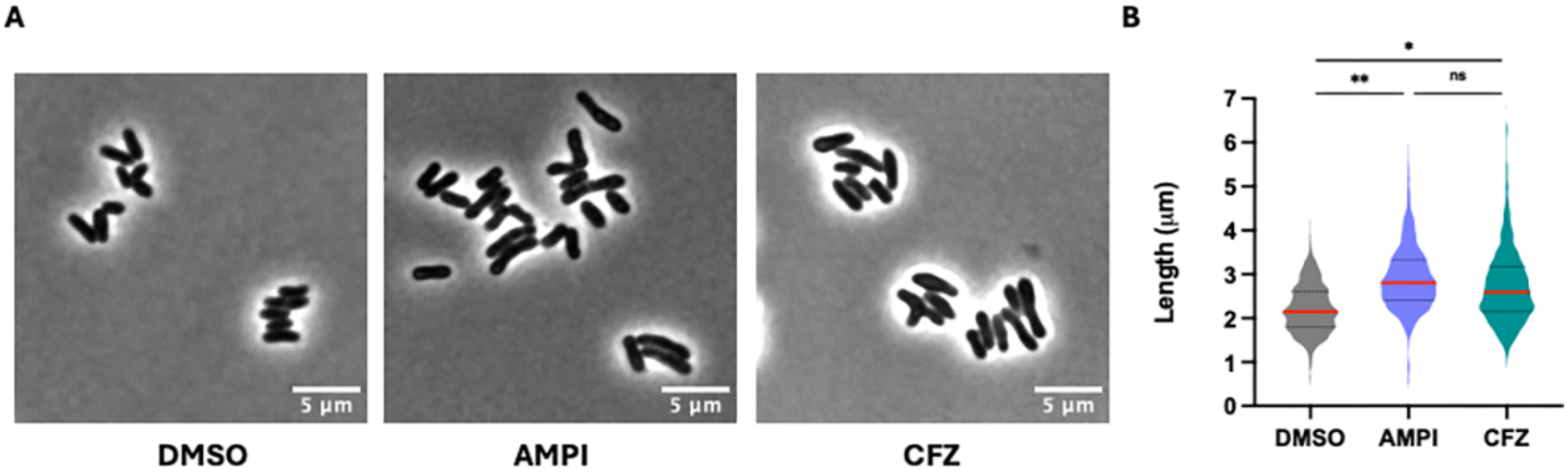

Figure S6. Comparison of *Cglu* morphologies exposed to clofazimine and ampicillin. *Cglu* cultures were inoculated at OD_{600}=1. After 2 h of growth, antibiotics were added and cultures were incubated for 18 h. Bacteria were treated with antibiotics following same protocol as the one used to generate the high-throughput image datasets (Materials and Methods). Images are representative of at least 2 independent experiments. (**A**) Phase contrast images were acquired for 100 ms. Scale bars, 5 µm. (**B**) Violin plots show the distribution of cell length (between bacterial cells treated with DMSO (n=1519), ampicillin (AMPI; n=1100) and clofazimine (CFZ; n=1253). Cohen's d: DMSO/AMP, **d=1.05; DMSO/CFZ, *d=0.72; AMPI/CFZ, ns=0.2.

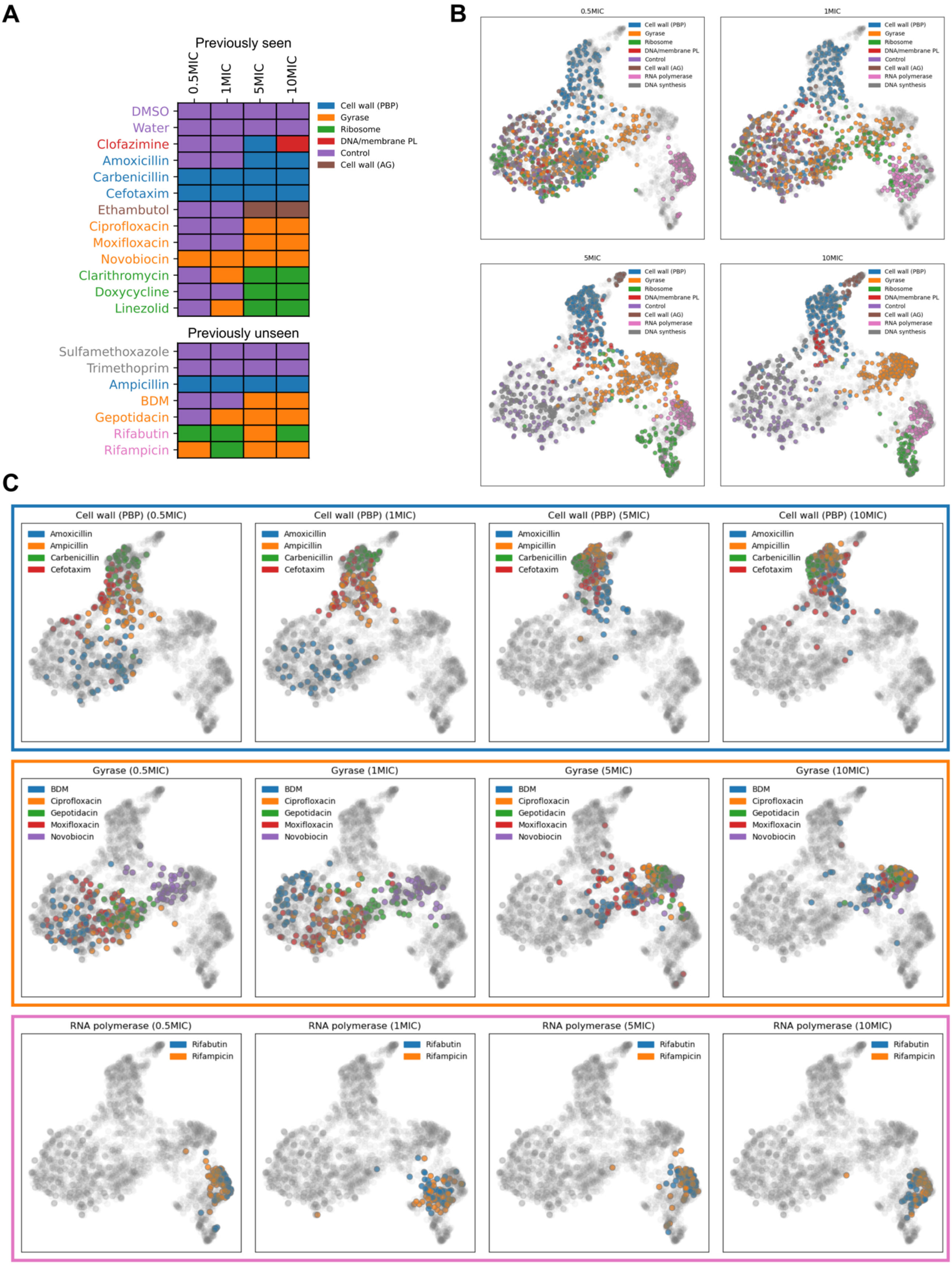

Figure S7. Concentration-dependent effects. (**A**) Well-level MoA predictions obtained from images of bacteria exposed to both previously seen and unseen antibiotics at four different concentrations on a hold-out

test plate with a DL model fine-tuned on images of bacteria exposed to antibiotics at 10xMIC. (**B, C**) Lower-dimensional projection of feature vectors from images of bacteria exposed to antibiotics at four concentrations was obtained with UMAP. Dots correspond to individual images and colours to the MoA of a given antibiotic. Dose-dependent latent trajectories can be observed with feature vectors tending to localise further away from control conditions with increasing concentration.

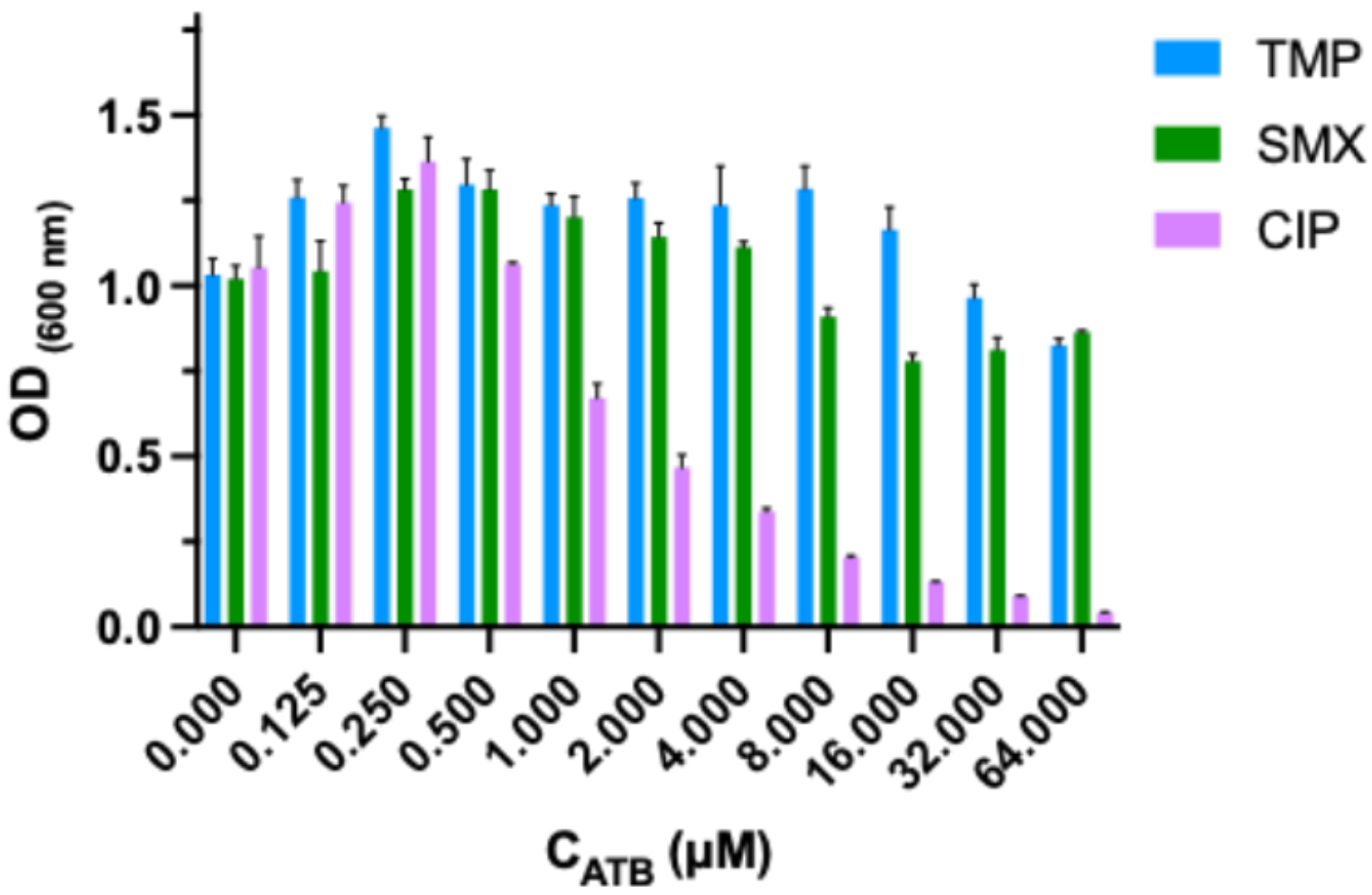

Figure S8. Trimethoprim and Sulfamethoxazole are inactive in *Cglu.* *Cglu* was inoculated at a final OD_{600}=0.2 in a 96-well plate and exposed to antibiotics (trimethoprim TMP, sulfamethoxazole SMX and ciprofloxacin CIP) at different concentrations (as indicated). Bacterial growth was recorded after 18 h and quantified by OD_{600} measurements. Bars indicate mean values with error bars corresponding to mean ± sd. Each concentration point corresponds to 3 replicates.

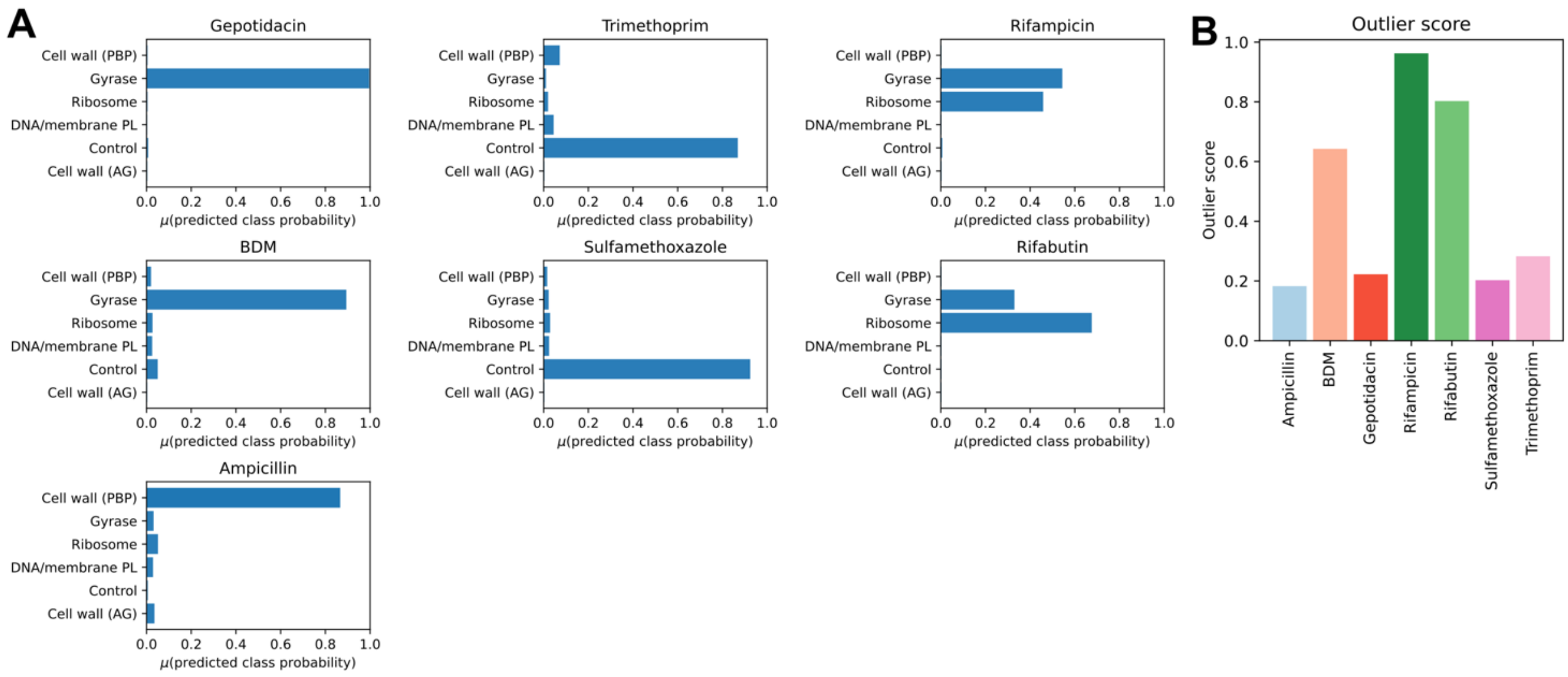

Figure S9. Predictions on previously unseen drugs and outlier scores. (**A**) Mean classification probabilities averaged across all FOVs are shown for each of the previously unseen drugs. (**B**) Outlier scores for previously unseen drugs obtained with a local outlier factor algorithm (Materials and Methods).

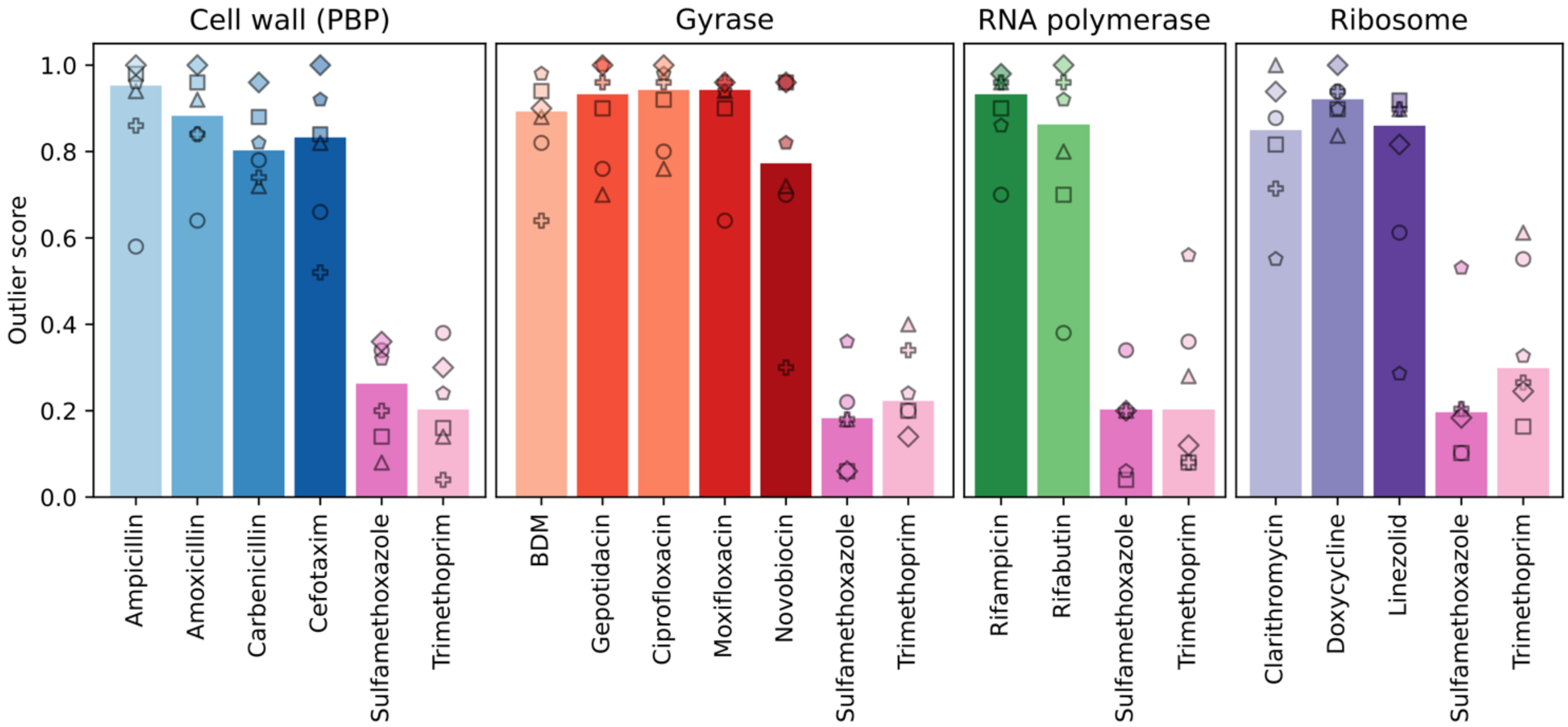

Figure S10. Outlier scores computed for drugs with a previously unseen MoA. Median outlier scores (Materials and Methods) for drugs from four different MoAs that were left out of the training data with symbols showing per-plate outlier scores.

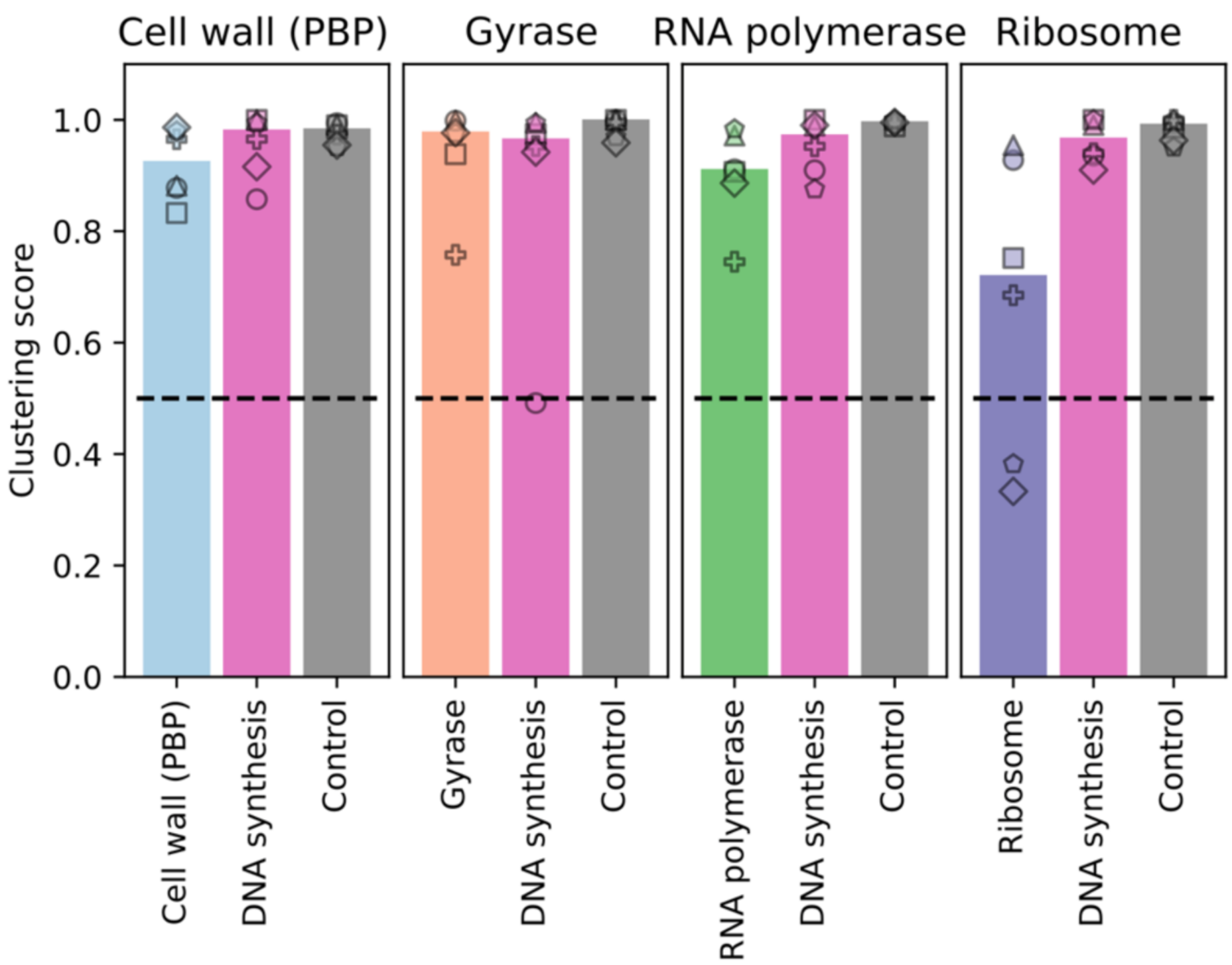

Figure S11. Clustering scores computed for drugs with a previously unseen MoA. Median clustering scores (Materials and Methods) for drugs from four different MoAs that were left out of the training data with symbols showing per-plate clustering scores. Dashed lines show the chance level.

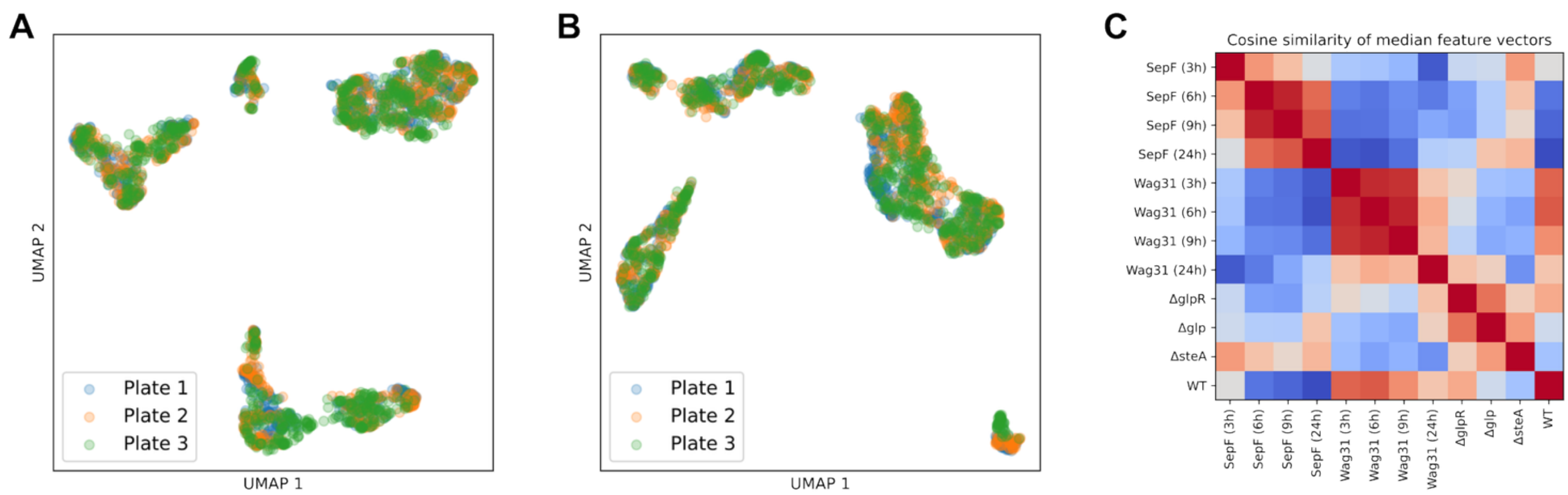

Figure S12. Replicate consistency and cosine similarity matrix. (**A,B**) UMAP of feature vectors coloured by plate showing good agreement across replicates for data shown in (**A**) Figure 4B and (**B**) Figure 5C. (**C**) Cosine similarity matrix computed on median feature vectors of mutants from which the adjacency matrix in Figure 5D is obtained.

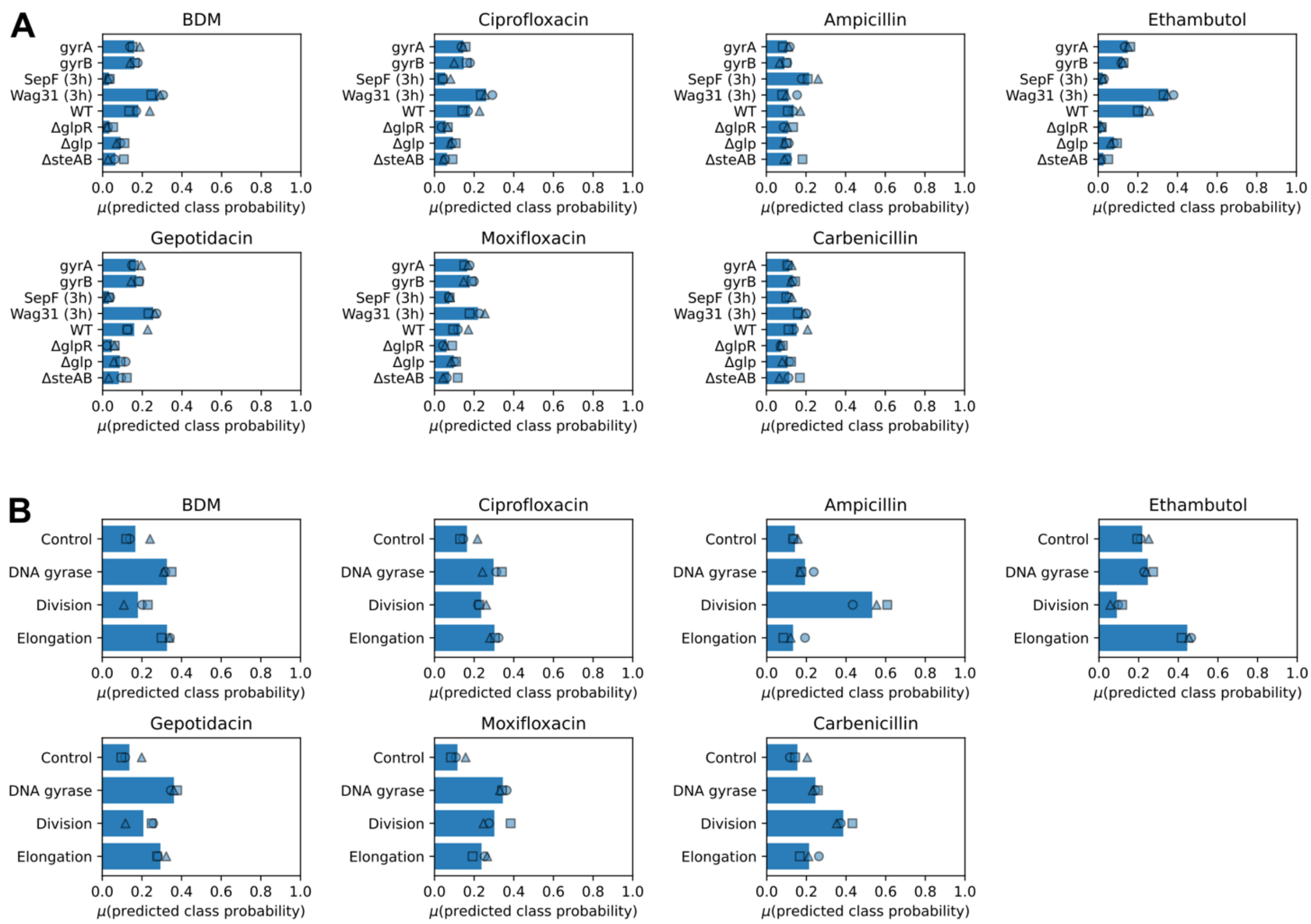

Figure S13. Disrupted pathway predictions on drugs with a random forest classifier trained on hand-crafted features. (**A, B**) Random forest classifiers were trained on hand-crafted features to directly predict the disrupted genes (**A**) or broad pathways (**B**) from feature vectors of mutants using two of the three available plates. Predictions on hand-crafted features derived from images of drug-treated bacteria were then obtained using a third hold-out test plate. In total, three models were trained and evaluated on three hold-out test plates with symbols showing per-replicate class probabilities.

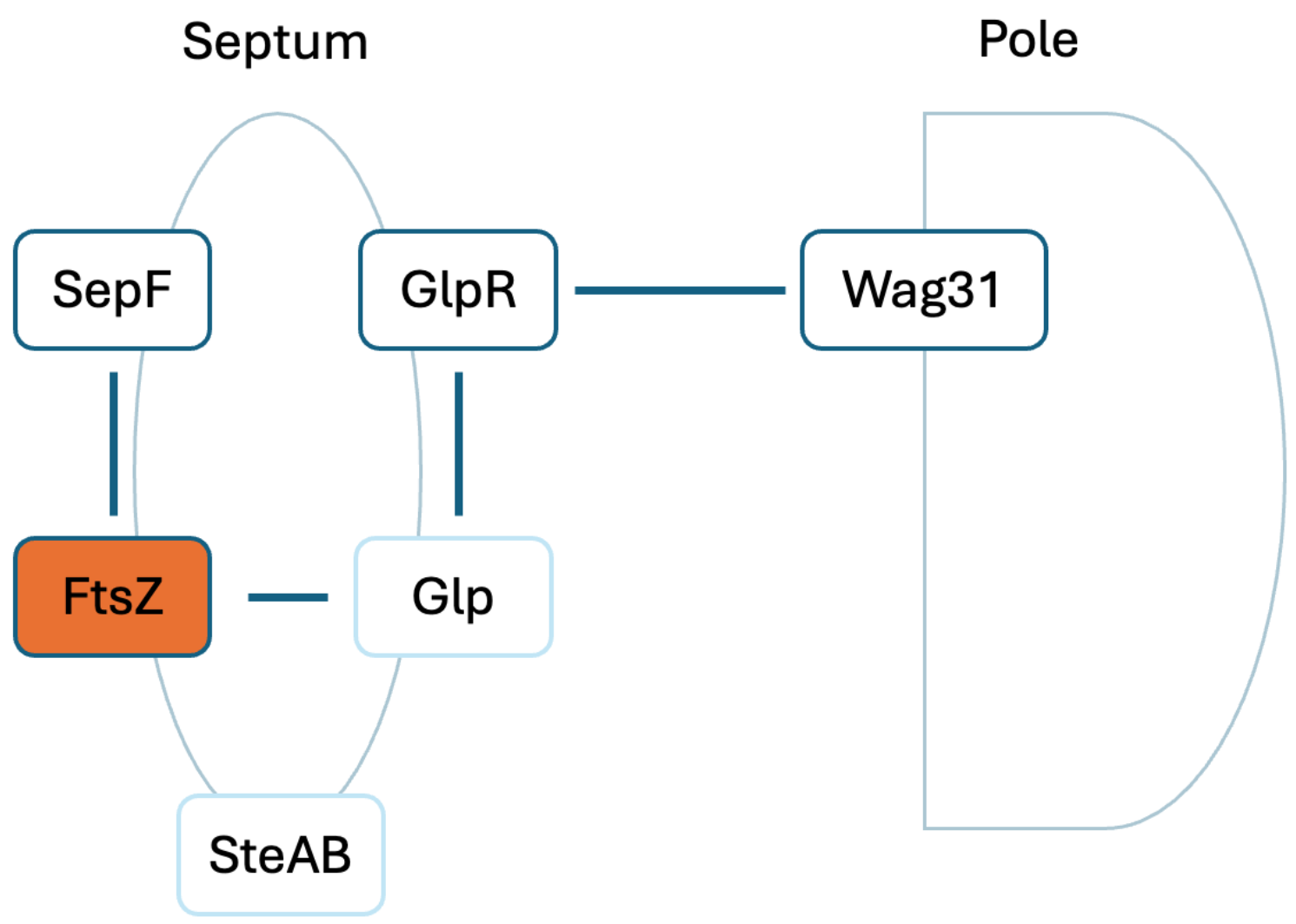

Figure S14: Current understanding of protein interactions for the cell cycle mutants used in Figure 5. Dark lines represent experimentally validated direct protein-protein interactions at the septum. Glp and SteAB mutants shown in light blue boxes exhibit elongated multiseptal phenotypes *(38, 39)*. FtsZ (orange, not included in the analysis) is the master organiser of cell division. The SepF mutant can be used as a proxy for abolishing the function of FtsZ, as in the absence of SepF, FtsZ cannot assemble at mid cell and initiate septum formation *(37)*. Septum formation is directly linked to cell wall biosynthesis. The cells depleted for SepF, SteAB or Glp are elongated *(38, 39).* GlpR is WT-like when analysed for length, width and FtsZ localization *(39)*. Wag31 depletion leads to cellular rounding. The 3h point is close to WT.

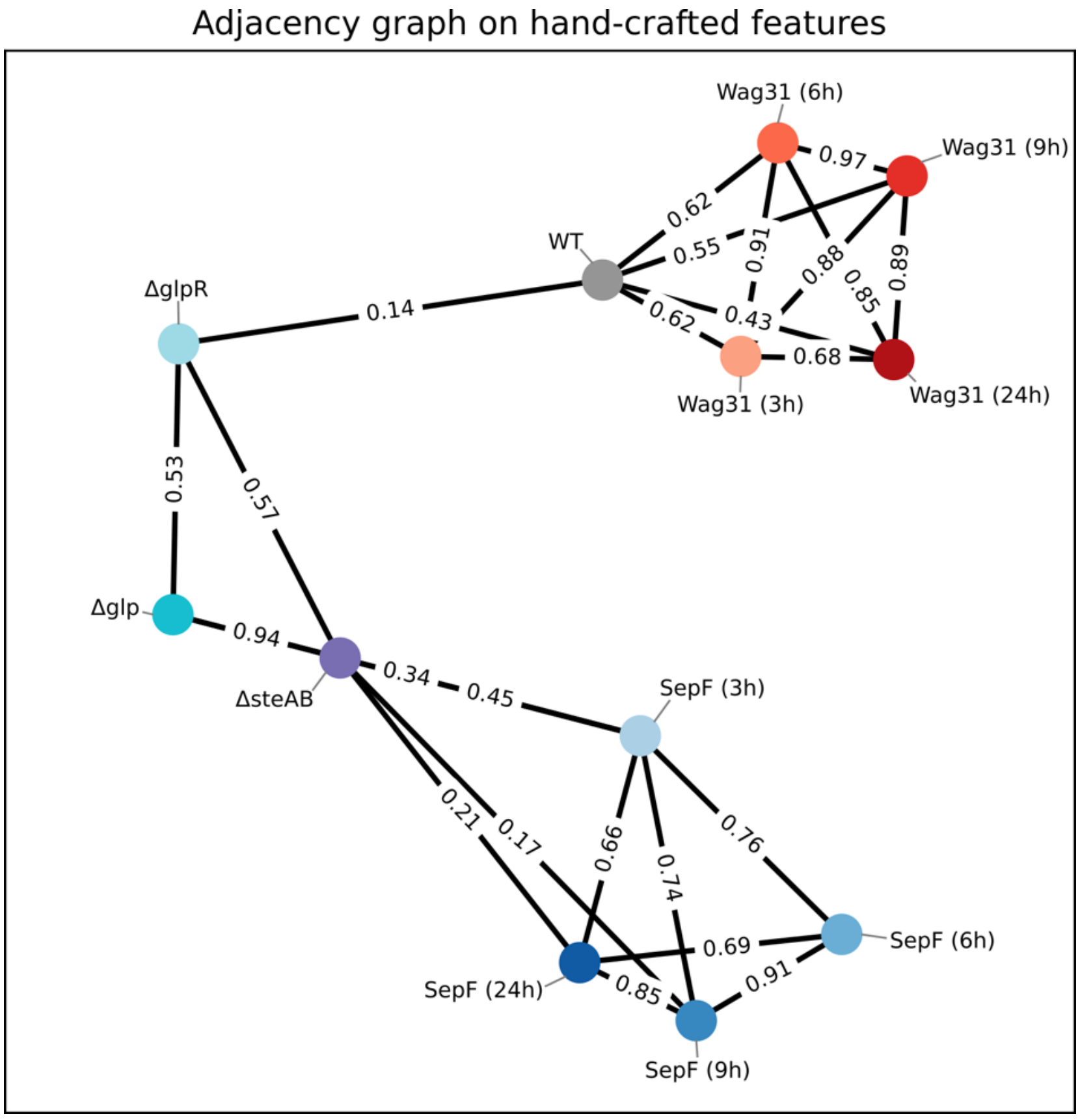

Figure S15. Adjacency graph of *Cglu* mutants disrupted in their cell cycle computed from hand-crafted features. Adjacency graph obtained from a cosine similarity matrix computed on median hand-crafted feature vectors and coloured by mutants showing connections between mutants with the most similar feature vectors. Values on edges correspond to cosine similarities between mutants. The threshold for the adjacency matrix was set such that each node was connected by at least one edge.

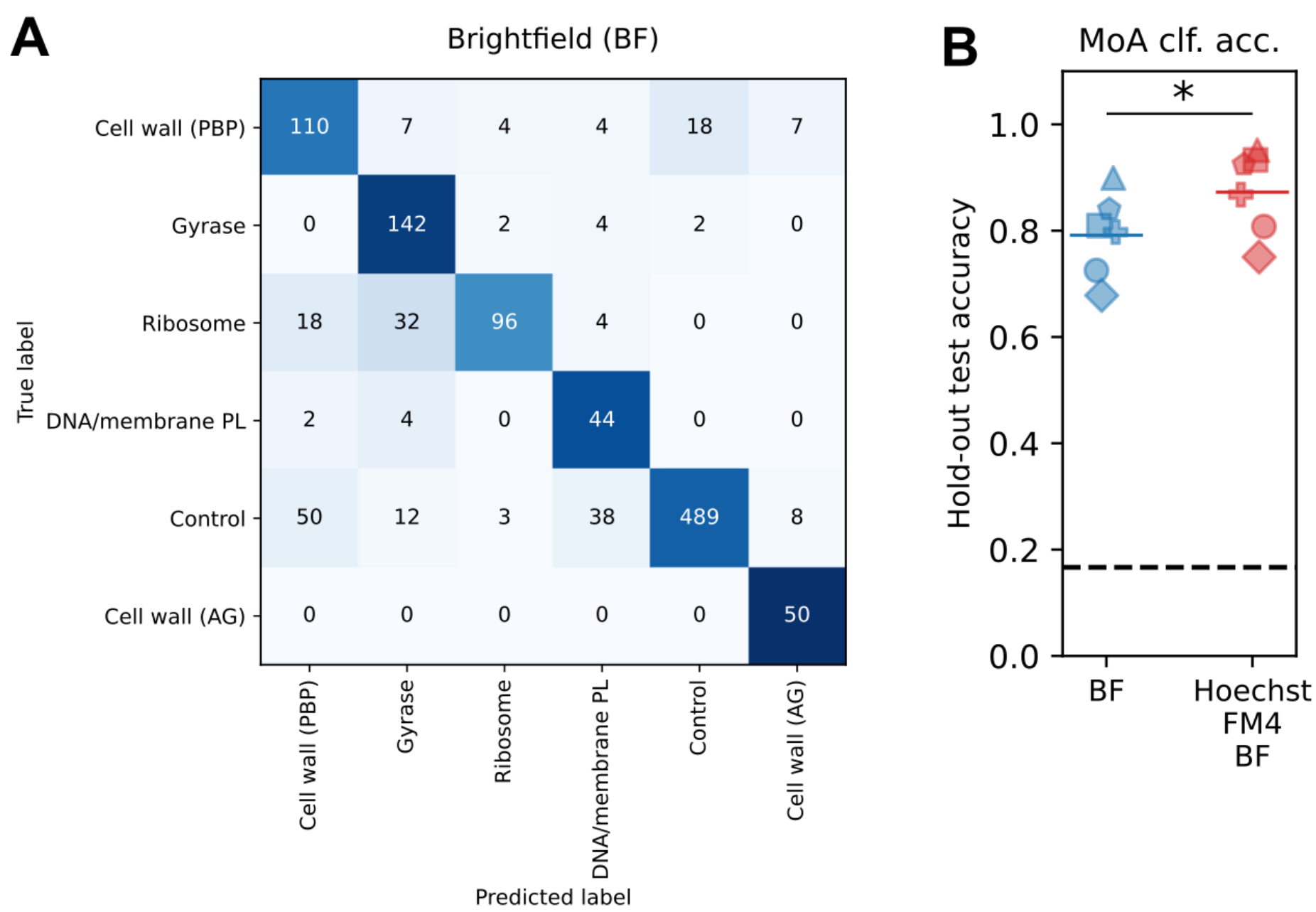

Figure S16. Model performance for different choices of imaging channels. (**A**) Confusion matrix on a hold-out test plate for a model only trained using the brightfield channel at 10xMIC. Values within the matrix indicate the number of FOVs. (**B**) Cross-validated MoA classification performance for models trained only using brightfield ('BF', blue) and brightfield in combination with Hoechst 33342 and FM4-64 ('Hoechst FM4 BF', red) at 10xMIC. Horizontal lines indicate mean classifications accuracies and symbols show accuracies for individual hold-out test plates. MoA classifications accuracies are 79.16±7.25% for brightfield-only and 87.25±7.27% for all channels. Statistical significance was computed with a two-sided Wilcoxon signed-rank test (p=0.031) for n=6 plates.

Table S1: Bacterial mutant strains

Strain	Genetic modification	Reference	Affected gene	Affected pathway
Cglu_WT	∅	-	∅	∅ wild-type rod-shaped
Cglu_P_{ino}-wag31	Conditional depletion	*(40)*	cg2361	Elongation defect (no elongation, ovoid to coccoid)
Cglu_P_{ino}-sepF	Conditional depletion	*(37)*	cg2363	Early division defect (no division, elongated to branched)
Cglu_P_{ino}-GA	Conditional depletion	*(35)*	cg0015	DNA segregation, diffuse nucleoid
Cglu_P_{ino}-GB	Conditional depletion	*(35)*	cg0007	DNA segregation defect, diffuse nucleoid
Cglu_Δglp	Knockout	*(39)*	cg1005	Division defect (multiseptal, elongated)
Cglu_ΔglpR	Knockout	*(39)*	cg1003	Wild-type rod-shaped
Cglu_ΔsteAB	Knockout	*(38)*	cg1603/ cg1604	Cell wall defect (multiseptal, elongated)

Supplementary auxiliary file: 'aeg6806_Suppl. Excel_seq1_v1.xlsx'
